# A Supervoxel-Based Method for Groupwise Whole Brain Parcellation with Resting-State fMRI Data

**DOI:** 10.3389/fnhum.2016.00659

**Published:** 2016-12-27

**Authors:** Jing Wang, Haixian Wang

**Affiliations:** Key Laboratory of Child Development and Learning Science of Ministry of Education, Research Center for Learning Science, Southeast UniversityNanjing, China

**Keywords:** resting-state fMRI, functional connectivity, whole brain parcellation, spectral clustering, supervoxel

## Abstract

Node definition is a very important issue in human brain network analysis and functional connectivity studies. Typically, the atlases generated from meta-analysis, random criteria, and structural criteria are utilized as nodes in related applications. However, these atlases are not originally designed for such purposes and may not be suitable. In this study, we combined normalized cut (Ncut) and a supervoxel method called simple linear iterative clustering (SLIC) to parcellate whole brain resting-state fMRI data in order to generate appropriate brain atlases. Specifically, Ncut was employed to extract features from connectivity matrices, and then SLIC was applied on the extracted features to generate parcellations. To obtain group level parcellations, two approaches named mean SLIC and two-level SLIC were proposed. The cluster number varied in a wide range in order to generate parcellations with multiple granularities. The two SLIC approaches were compared with three state-of-the-art approaches under different evaluation metrics, which include spatial contiguity, functional homogeneity, and reproducibility. Both the group-to-group reproducibility and the group-to-subject reproducibility were evaluated in our study. The experimental results showed that the proposed approaches obtained relatively good overall clustering performances in different conditions that included different weighting functions, different sparsifying schemes, and several confounding factors. Therefore, the generated atlases are appropriate to be utilized as nodes for network analysis. The generated atlases and major source codes of this study have been made publicly available at http://www.nitrc.org/projects/slic/.

## Introduction

The functional organization of human brain could be characterized by brain networks (Sporns et al., 2005; Bullmore and Sporns, 2009). It is therefore of great significance to construct functional connectivity networks at system level for the brain. To fulfill this purpose, nodes should be defined in prior (Wig et al., 2011). The nodes could be defined at voxel scale or whole brain scale in extreme conditions. When nodes are defined at voxel scale, the resultant network would be computationally expensive, vulnerable to noise, and difficult to be interpreted due to the intrinsic property of fMRI data (Craddock et al., 2012). On the other hand, when nodes are defined at whole brain scale, the resultant network might be too coarse to reveal some concealed connectome characteristics (de Reus and Van den Heuvel, 2013; Shen et al., 2013). An intermediate approach, i.e., to parcellate the brain into a specific number of regions and treat each of them as a node, could alleviate the above problems. However, no consensus has been reached on how the brain should be parcellated.

In existing studies, some typical ways to generate whole brain atlases are based on meta-analysis, random criteria, structural criteria, and functional connectivity (Wig et al., 2011; de Reus and Van den Heuvel, 2013; Fornito et al., 2013; Sporns, 2013; Stanley et al., 2013). Parcellation by meta-analysis of peak activations (Dosenbach et al., 2010; Power et al., 2011) is laborious and inaccurate, and lacks reproducibility (Craddock et al., 2012). Parcellation based on random criteria (Hagmann et al., 2008; Zalesky et al., 2010) serves as an effective tool to study network statistics at different node scales. Unfortunately, its resultant atlases do not contain any neurobiological meanings. Hence, their usages are limited. Parcellation based on structural criteria such as cytoarchitecture (Zilles and Amunts, 2010), myelin content (Glasser and Van Essen, 2011), and tractography (Behrens et al., 2003) provides standardized atlases which have been widely applied in various studies. Since brain function is closely related with brain structure (Honey et al., 2009), it is expected that the structurally defined brain atlas contains functional meanings and could be used to construct functional connectivity networks. Strictly speaking, however, structural homogeneity does not necessarily guarantee functional homogeneity, i.e., some functionally heterogeneous time courses might be mixed in a single node in a structurally defined brain atlas, thus greatly affecting network estimation and some topological attributes of the brain network (Wang et al., 2009; Smith et al., 2011). The above limitations necessitate the application of resting-state functional connectivity (RSFC) in whole brain parcellation.

The basic idea of connectivity-based parcellation is to aggregate voxels with similar connectivity patterns into clusters. Previous studies concerning RSFC-based parcellation mainly focus on subdividing a region of interest (ROI) rather than the whole brain. Plenty of clustering algorithms have been applied, including but not limited to, independent components analysis (ICA; McKeown et al., 1998; Beckmann et al., 2005; Damoiseaux et al., 2006; De Luca et al., 2006; Smith et al., 2009), hierarchical clustering (Goutte et al., 1999; Cordes et al., 2002; Stanberry et al., 2003; Salvador et al., 2005; Mumford et al., 2010; Thirion et al., 2014), spectral clustering (van den Heuvel et al., 2008; Shen et al., 2010, 2013; Craddock et al., 2012), K-means (Kim et al., 2010; Kahnt et al., 2012; Chang et al., 2013), region growing (Lu et al., 2003; Heller et al., 2006), von Mises-Fisher distributions (Yeo et al., 2011; Ryali et al., 2013), self-organizing maps (Peltier et al., 2003), Infomap (Power et al., 2011), and modularity detection (Chang et al., 2014). Some studies combine different clustering algorithms together to perform parcellation. For example, Filzmoser et al. (1999) proposed a hierarchical K-means approach; Bellec et al. (2006) combined region growing with hierarchical clustering; and Bellec et al. (2010) combined region growing, K-means, and hierarchical clustering.

A special case of RSFC-based parcellation is to parcellate the cortical surface rather than the brain volume (Cohen et al., 2008; Nelson et al., 2010; Wig et al., 2014; Gordon et al., 2016). It is a convention in surface-based analysis where the brain is treated as a 2D sheet rather than a 3D volume. In such analysis, the abrupt transitions in RSFC patterns are used to identify putative boundaries between cortical areas, known as the boundary mapping approach (Cohen et al., 2008). Other methods such as hierarchical clustering (Blumensath et al., 2013) and spectral clustering (Parisot et al., 2016) also could be applied to parcellate the cortical surface.

Moreover, some related studies are applying clustering algorithms on diffusion MRI data to perform connectivity-based parcellation (Behrens et al., 2003; Johansen-Berg et al., 2004). Although diffusion MRI data is quite different from resting-state fMRI data, the clustering algorithms could be implemented similarly once connectivity is defined. For example, K-means (Johansen-Berg et al., 2004; Klein et al., 2007), spectral clustering (Venkataraman et al., 2009; Fan et al., 2014, 2016; Zhang et al., 2014), hierarchical clustering (Gorbach et al., 2011; Moreno-Dominguez et al., 2014), and some other clustering algorithms (Behrens et al., 2003; Jbabdi et al., 2009) are applied to perform structural connectivity-based parcellation.

Recently, Glasser et al. (2016) used a semi-automated approach to parcellate the human cerebral cortex into 180 areas per hemisphere based on multi-modal magnetic resonance images. Specifically, the parcellation was determined both by a clustering algorithm and by experienced neuroanatomists; it utilized information related to four areal properties including cortical architecture, function, connectivity, and topography. The study represents a new milestone in the research area of brain parcellation.

As discussed above, parcellation approaches could be implemented in different ways, e.g., on diffusion MRI data or on resting-state fMRI data, on the cortical surface or on the brain volume, on a ROI or on the whole brain. Among these studies, only few (Craddock et al., 2012; Blumensath et al., 2013; Shen et al., 2013; Moreno-Dominguez et al., 2014; Thirion et al., 2014; Parisot et al., 2016) generate whole brain atlases at multiple granularities. The potential to extend other clustering methods to fulfill the purpose might be limited by high computational complexity due to huge data size and complicate parameters. This paper focuses on parcellating the whole brain based on RSFC in order to reveal the functional organization of the human brain. We combined Ncut and SLIC to address this task.

Ncut (Shi and Malik, 2000) is a graph theoretic approach. It has many advantages over other clustering algorithms such as being easy to implement, being robust to outliers, and achieving good clustering performances (von Luxburg, 2007). It is theoretically designed to partition a graph into two disjoint sets. In order to obtain multiple clusters, there are different methods including bipartitioning the graph recursively (Shi and Malik, 2000), applying K-means on the eigenvectors of the graph Laplacian (Ng et al., 2002; von Luxburg, 2007), and the multiclass spectral clustering (MSC) algorithm (Yu and Shi, 2003). All of these methods treat the eigenvectors of the graph Laplacian as features for further clustering. These features contain less redundancy and are less sensitive to noises than the original data. From this viewpoint, Ncut could be regarded as a dimensionality reduction and feature extraction algorithm that serves as a necessary step prior to clustering. As for applications of Ncut in brain parcellation, Fan et al. (2014) and Zhang et al. (2014) applied K-means on the features extracted by variants of Ncut, Craddock et al. (2012) applied the MSC algorithm that integrated Ncut with a clustering algorithm, and Shen et al. (2013) extended MSC to a formulation known as the multigraph K-way spectral clustering (MKSC) algorithm which processed multiple subjects simultaneously. Both MSC and MKSC wrap a feature extraction step in the clustering algorithm. For clarity, the corresponding clustering algorithms without the feature extraction step are referred to as MSC and MKSC in the following paper.

SLIC is an important supervoxel method. The supervoxel method originates from its 2D counterpart, the superpixel method (Achanta et al., 2012). The superpixel method has drawn increasing interest in the field of computer vision in recent years. Its basic idea is to partition an image into perceptually meaningful patches, termed superpixels, wherein the pixels have similar intensity or colors. This method could effectively extract image structure and capture image redundancy. Hence, it could be used as an effective segmentation or clustering algorithm. Among different superpixel methods, SLIC is a very popular one due to its simplicity, effectiveness, and good segmentation performances (Achanta et al., 2012). Another important advantage of SLIC is that it is straightforward to be extended to a supervoxel generation method (Lucchi et al., 2012), which makes it suitable to parcellate the brain volume. Previously, we had presented a study in Wang et al. (2016) by directly applying the SLIC algorithm on resting-state fMRI time series to perform whole brain parcellation. Only the individual subject level parcellation was investigated in that study.

In the current study, we incorporated Ncut and SLIC to perform whole brain parcellation. To generate group level parcellations, two approaches, i.e., the mean SLIC and two-level SLIC approaches, were proposed. To evaluate the performance of the proposed approaches, we compared them with three state-of-the-art Ncut-based approaches under different evaluation metrics. The influences of several confounding factors were carefully investigated in order to demonstrate the rationality of the proposed approaches.

## Materials and methods

### Participants and imaging data acquisition

Forty healthy Chinese college students (19 females and 21 males, 19–27 years old, mean age = 22.8 years, *SD* = 1.37) were recruited for the study. All of them had normal or corrected-to-normal vision and reported no history of psychiatric or neurological diseases. Two additional participants were recruited but excluded from analysis due to large head motion (>2 mm and 2°). Written informed consent was obtained from each participant after explaining the study purpose and procedure. This study was approved by the Institutional Review Boards of Beijing Normal University.

All structural and resting-state functional MRI images were collected on a 3-Tesla Siemens Trio system. High-resolution T1-weighted structural images were acquired with a Magnetization Prepared Rapid Acquisition Gradient-Echo (MPRAGE) sequence: repetition time (TR) = 2530 ms, echo time (TE) = 3.39 ms, inversion time (TI) = 1100 ms, flip angle (FA) = 7°, field of view (FOV) = 256 × 256 mm^2^, slices = 144, thickness = 1.33 mm, voxel size = 1.3 × 1.0 × 1.3 mm^3^. Resting-state fMRI images were acquired using a Gradient Echo type Echo Planar Imaging (GRE-EPI) sequence: TR = 2000 ms, TE = 30 ms, FA = 90°, in-plane resolution = 64 × 64, FOV = 200 × 200 mm^2^, thickness = 3.5 mm, slice gap = 0.7 mm, acquisition voxel size = 3.1 × 3.1 × 3.5 mm^3^. Thirty-three slices were used to cover the whole brain. Two hundred volumes of resting-state fMRI data were collected for each subject. During the resting-state scanning, all subjects were instructed to close their eyes and relax.

### Preprocessing

The resting-state fMRI data was preprocessed by the Data Processing Assistant for Resting-State fMRI (DPARSF) (Yan and Zang, 2010) which was built on Statistical Parametric Mapping (SPM; Friston et al., 1994). Structural and functional data were first visually inspected for imaging errors such as signal dropouts and ghosting. No images were removed. The first 10 volumes were discarded to allow signal stabilization. Then all functional time series were processed by slice timing correction and motion correction. The structural data was coregistered to the mean functional data so that the mutual information between them was maximized. The coregistered structural data was segmented into gray matter (GM), white matter (WM), and cerebrospinal fluid (CSF) using the default tissue probability maps in SPM as priors. The Diffeomorphic Anatomical Registration using Exponentiated Lie algebra (DARTEL) toolbox (Ashburner, 2007) was used to compute transformations between individual native space and Montreal Neurological Institute (MNI) space. Finally, the slice-time corrected and motion corrected functional data was processed by the following steps: normalized to the MNI space at 4 × 4 × 4 mm^3^ resolution by DARTEL; spatially smoothed by a Gaussian kernel with a full width at half maximum (FWHM) of 6 mm; linear detrended; bandpass filtered with passband 0.01~0.08 Hz; denoised by regressing out nuisance covariates including six head motion parameters, autoregressive models of motion (the Friston 24-parameter model; Friston et al., 1996; Yan et al., 2013), and the mean time courses of WM signal and CSF signal.

### Parcellation approaches

The proposed parcellation approaches included two group level clustering approaches that were achieved by combining Ncut and SLIC. To apply these approaches, a weight matrix should be defined in prior. We conducted a normalization step before defining the weight matrix in order to facilitate subsequent calculation.

#### Normalization

After preprocessing the resting-state fMRI data, we focused on gray matter only, to be consistent with (Craddock et al., 2012). It is by no means indicating that other brain regions such as the white matter is less important in fulfilling brain activities (Gawryluk et al., 2014), and our approaches could certainly be extended to include those regions. The choice was made mainly based on the methodological consideration of reducing the computation time. For the *i*th voxel in the gray matter mask, denote its time course as *v*_*i*_, *i* = 1, 2, …, *N*. Each time course within the gray matter was normalized to have zero mean and unit length (Shen et al., 2010). Specifically, for a raw time course *v* in the gray matter, it was normalized to be

$$\begin{array}{l} \frac{v\;-\;\bar{v}}{{\left\|v\;-\;\bar{v}\right\|}_{2}}, \end{array}$$

where $\bar{v}$ denotes the mean value of this time course, ‖·‖_2_ denotes L2-norm of a vector. After normalization, the Pearson correlation coefficient between two time courses *v*_*i*_ and *v*_*j*_ equals their dot product

$$\begin{array}{l} corr\left(v_{i},\;v_{j}\right)=v_{i}\cdot v_{j}. \end{array}$$

Assume *V* is the resting-state fMRI data wherein each row is a normalized time course. Then the correlation matrix could be calculated by *VV*^*T*^. This trick helps to reduce computational time. In addition, it is easy to validate that

$$\begin{array}{l} corr\left(v_{i},\;v_{j}\right)=1-{\left\|v_{i}-v_{j}\right\|}_{2}^{2}/2. \end{array}$$

This relationship bridges the gap between the Pearson correlation coefficient and the Euclidean distance, making it reasonable to use either of them as the similarity measure when constructing the weight matrix.

#### Individual subject level weight matrix

Three typical definitions of the weight matrix are described as follows. For the *i*th voxel in the gray matter mask, denote its coordinate in the MNI space as *u*_*i*_, *i* = 1, 2, …, *N*. By adopting the original formulation in (Shi and Malik, 2000), the weight on the edge connecting voxels *i* and *j* could be defined as

$$w_{ij}=\{\begin{array}{l} e^{-\frac{{\left\|v_{i}-v_{j}\right\|}_{2}^{2}}{\sigma_{v}^{2}}-\frac{{\left\|u_{i}-u_{j}\right\|}_{2}^{2}}{\sigma_{u}^{2}}}\text{ if}{\left\|u_{i}-u_{j}\right\|}_{2}\;\leq \;r\\ \text{0 otherwise} \end{array},$$

where σ_*v*_, σ_*u*_ and *r* are three tuning parameters. In (Craddock et al., 2012), the weight was defined by the Pearson correlation coefficient with a spatial constraint and a hard threshold

$$w_{ij}=\{\begin{array}{l} corr(v_{i},\;v_{j})\text{ if}{\left\|u_{i}-u_{j}\right\|}_{2}\leq \;\sqrt{3}\;and\;corr(v_{i},\;v_{j})\;=0.5\\ \text{0 otherwise} \end{array}.$$

The two constraints were applied to make the weight matrix sparse, and to exclude the negative and weak correlations. A potential change of the above formula is to replace the correlation between two fMRI time series with the correlation between their connectivity profiles (Cohen et al., 2008), but this change would not improve the parcellation performance according to studies (Craddock et al., 2012; Blumensath et al., 2013). Hence, it was not applied. In (Shen et al., 2010, 2013), the weight was defined by a Gaussian function of the Euclidean distance

$$\begin{array}{l} w_{ij}=e^{-\frac{{\left\|v_{i}-v_{j}\right\|}_{2}^{2}}{\sigma^{2}}}, \end{array}$$

where σ was set to be the median of all functional distances. To make this weight matrix sparse, only a certain number of the largest values in each row and each column were reserved.

Based on the above definitions, we conclude that there are three principles that should be followed in defining the weight matrix. First, the weighting function should be negatively related to the functional distance and the spatial distance. It is natural since the weight should reflect the likelihood that two voxels belong to one cluster (Shi and Malik, 2000), and the likelihood tends to be negatively related to the two distances. Strictly speaking, some weighting functions (Shen et al., 2010; Craddock et al., 2012) do not involve the spatial distance. They are variants of the weight in Shi and Malik (2000). Second, the weight matrix should be sparse in order to reduce computational burden. A full weight matrix is usually unmanageable with limited computational capacities. Third, the weight matrix should be symmetrical since parcellation approaches are generally applied on undirected networks.

**Figure 1:**
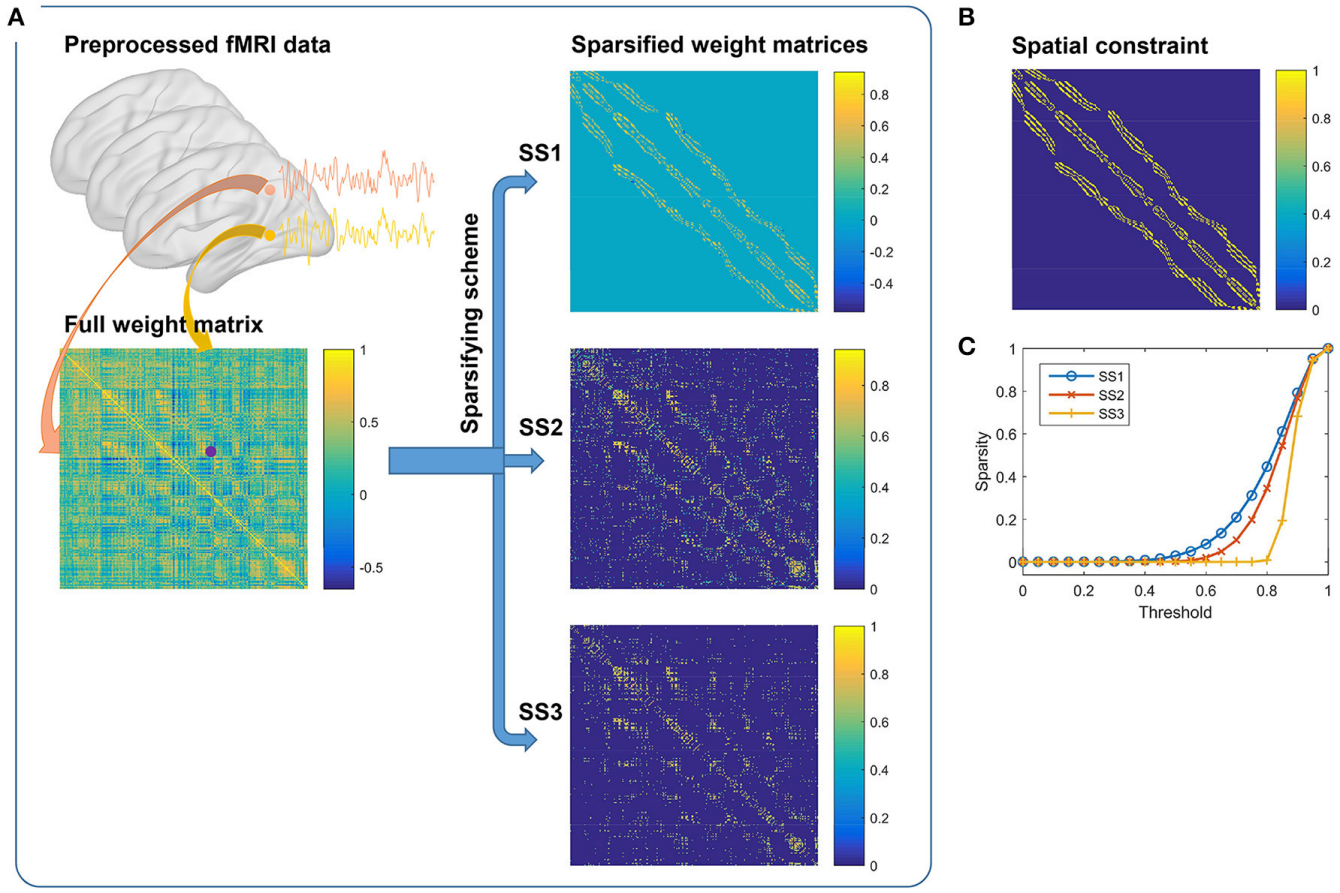
**(A)** Generating weight matrices by the three sparsifying schemes. It consists of two steps. The first step is to calculate the full weight matrix from the preprocessed fMRI data by correlating two fMRI time series. The second step is to apply one of the three different sparsifying schemes on the full weight matrix. **(B)** The spatial constraint. **(C)** By pulling together the non-zero elements in each sparsified weight matrix, we could obtain a new vector. Sparsity is defined by the ratio of zero elements in the vector when different thresholds are set to the vector. The results are averaged across subjects.

It is notable that the definition of the weight matrix could be decomposed into two steps. The first step is to choose a weighting function, and the second step is to sparsify the weight matrix. This procedure is illustrated in Figure 1A wherein a downsampled fMRI data is used for clarity. In our study, we chose the Pearson correlation coefficient as the default weighting function. The Gaussian kernel function and a constant function were tested for comparison later. For the sparsifying scheme (SS), three choices were tried. They were, applying a spatial constraint to the weight matrix, reserving a certain number of the largest values in each row and each column of the weight matrix, and setting a threshold to the weight matrix. The three sparsifying schemes are referred to as SS1, SS2, and SS3 for short in order. All of the generated weight matrices follow the three principles in defining the weight matrix.

To make the three sparsifying schemes comparable, we tried to ensure that they had similar sparse rates. In the first sparsifying scheme, the spatial constraint was chosen to be the 26-connected neighborhood (Craddock et al., 2012), as shown in Figure 1B. We calculated a sparse rate based on this scheme and applied it to the other two schemes. For the second scheme, the largest 17 values in each row and each column of the weight matrix were reserved, which would lead to a similar sparse rate. For the third scheme, the exact threshold was calculated based on the raw weight matrix and the sparse rate. To examine a sparsified weight matrix, we extracted the non-zero elements from it to form a new vector and calculated the sparse rate after applying different thresholds to this vector. Figure 1C shows the average sparse rates of the three sparsifying schemes. The reserved elements were generally larger than 0.5 and smaller than 1.0. Therefore, these weight matrices were different from the trivial weight matrix used for random parcellation in Craddock et al. (2012). The first two sparsifying schemes were closely related to those in Craddock et al. (2012) and Shen et al. (2013), respectively. Hence, we expected them to obtain similar results as in the previous studies.

Among the above definitions of the weight matrix and many other alternatives (Cheng et al., 2014), which one is the best still remains to be an open problem. It is often difficult to pick a suitable weighting function (Shi and Malik, 2000), and there does not exist a theoretical relationship between the choice of weight matrix and the clustering result (von Luxburg, 2007). Therefore, it is suggested that more attention should be placed on designing a clustering algorithm that could achieve stable performances with different weights. Ncut has an evident advantage in this respect according to the studies.

#### Ncut

After constructing the weight matrix, Ncut was employed to extract features and reduce data dimensionality. We briefly review Ncut (Shi and Malik, 2000) as follows. Denote fMRI data by an undirected weighted complete graph *G* = (*V, E*), wherein the nodes *V* correspond to the voxels in fMRI data and an edge in *E* is formed with a weight between a pair of voxels. In the binary case when the graph is intended to be partitioned into two disjoint clusters *A* and *B*, Ncut minimizes the following criterion

$$\begin{array}{l} Ncut\left(A,B\right)=\frac{cut\left(A,B\right)}{assoc\left(A,V\right)}+\frac{cut\left(A,B\right)}{assoc\left(B,V\right)}, \end{array}$$

where *cut*(*A, B*) is the sum of weights on edges connecting voxels in *A* to voxels in *B*, *assoc*(*A, V*) is the sum of weights on edges connecting voxels in *A* to all voxels in the graph, and *assoc*(*B, V*) is similarly defined. The criterion minimizes the between-cluster similarity and maximizes the within-cluster similarity simultaneously. This optimization problem is NP-hard. Fortunately, an approximate discrete solution for it is available by solving a generalized eigenvalue problem. To be specific, let *W* be an *N* × *N* symmetrical weight matrix with *W*(*i, j*) = *w*_*ij*_, *D* be an *N* × *N* diagonal matrix with

$$\begin{array}{l} D\left(i,i\right)=\underset{j\;=1}{\overset{N}{\sum }}w_{ij}, \end{array}$$

and then the generalized eigenvalue problem is given by

$$\begin{array}{l} \left(D-W\right)y=\lambda Dy, \end{array}$$

where *y* is the indicator vector to be solved. This problem could be transformed into a standard eigensystem

$$\begin{array}{l} D^{-\frac{1}{2}}\left(D-W\right)D^{-\frac{1}{2}}z=\;\lambda z, \end{array}$$

where $z=D^{\frac{1}{2}}y.\;D^{-\frac{1}{2}}\left(D-W\right)D^{-\frac{1}{2}}$ is known as the normalized graph Laplacian matrix. Since this matrix is theoretically rank deficient, a small positive regularization term should be added to it prior to eigendecomposition in practice. Once the eigenvector *z* is obtained by eigendecomposition, the indicator vector *y* could be calculated by $y\;=\;D^{-\frac{1}{2}}z$, and then it is further normalized to have unit norm. For more details of the Ncut algorithm, please refer to (Shi and Malik, 2000; von Luxburg, 2007).

Conventionally, the diagonal elements of the weight matrix *W* are set to be zeros (Zhang and Horvath, 2005). In practice, we followed this convention except for the third sparsifying scheme. This scheme might generate empty rows and columns in the weight matrix since a threshold is set globally to the weight matrix. Then there might be zero elements in the diagonals of *D*, and they would cause the division by zero errors when calculating $D^{-\frac{1}{2}}$. To fix the problem, we set the diagonal elements in the empty rows and columns to be ones. We had also tried to set all diagonal elements to be ones and obtained nearly the same clustering performances. Therefore, the parcellation approaches are not sensitive to a slight adjustment of the diagonal elements.

To parcellate the brain into *K* clusters, *K* indicator vectors corresponding to the *K* smallest non-trivial eigenvalues (>10^−4^) were computed. These indicator vectors constitute a feature matrix *X* of size *N* by *K* wherein each row is a feature corresponding to a voxel and each column is an indicator vector. The feature matrix was then input into different clustering algorithms, i.e., MSC, MKSC, and SLIC, to do clustering. For MSC and MKSC, we followed the algorithm procedure in Yu and Shi (2003) and Shen et al. (2013), respectively. For SLIC, the algorithm procedure will be described in the following section.

#### SLIC

After extracting features by Ncut, SLIC (Achanta et al., 2012) was applied on the features to perform clustering. SLIC is actually an adaptation of K-means. Two important differences between SLIC and K-means are that SLIC limits the search space to the neighborhood of a cluster center and creates a unified distance metric by integrating the spatial distance and the intensity distance. The algorithm procedure of SLIC is stated as follows. To parcellate the brain into *K* clusters, we first initialized *K* cluster centers. Let $S=\sqrt[{3}]{N/K}$, for each voxel in the 3*S* × 3*S* × 3*S* region around a cluster center, a distance between the voxel and the cluster center was calculated. This distance was assigned to the voxel as a measure to judge which cluster it should belong to. If the distance decreased comparing to the result in the previous iteration, then associated the voxel to the current cluster center. This procedure was repeated for all cluster centers. Once completed, the features and coordinates of the voxels within each new cluster were averaged separately to represent the feature and coordinate of the new cluster center correspondingly. The above assignment and update steps were repeated until the change of the cluster centers was lower than a certain threshold. Table 1 summarizes the algorithm procedure. Note that the search space was enlarged from the traditional 2*S* × 2*S* × 2*S* region to the 3*S* × 3*S* × 3*S* region in this study because the brain shape is irregular so that the traditional search region is too small to capture some border voxels. This enlargement made little changes to the parcellation results other than capturing the border voxels. Other shapes of search space were not tried since the cubic search space was adequate.

**Table 1:** **The algorithm procedure of the SLIC supervoxel algorithm**.

**Input:** the features extracted by Ncut and the initialized cluster number. **Output:** the cluster labels.
Initialize the cluster centers. Initialize label l(*i*) = −1 for each voxel *i*. Initialize distance d(*i*) = ∞ for each voxel *i*. **while** not converged **do** ** for** each cluster center *C*_*k*_ **do** **for** each voxel i in the 3*S* × 3*S* × 3*S* region around *C*_*k*_ **do** Compute the unified distance D between *C*_*k*_ and *i*. **if** *D* < *d*(*i*) **then** Set *l*(i) = k. Set *d*(*i*) = D. **end if** **end for** **end for** Compute new cluster centers. **end while**

**Figure 2:**
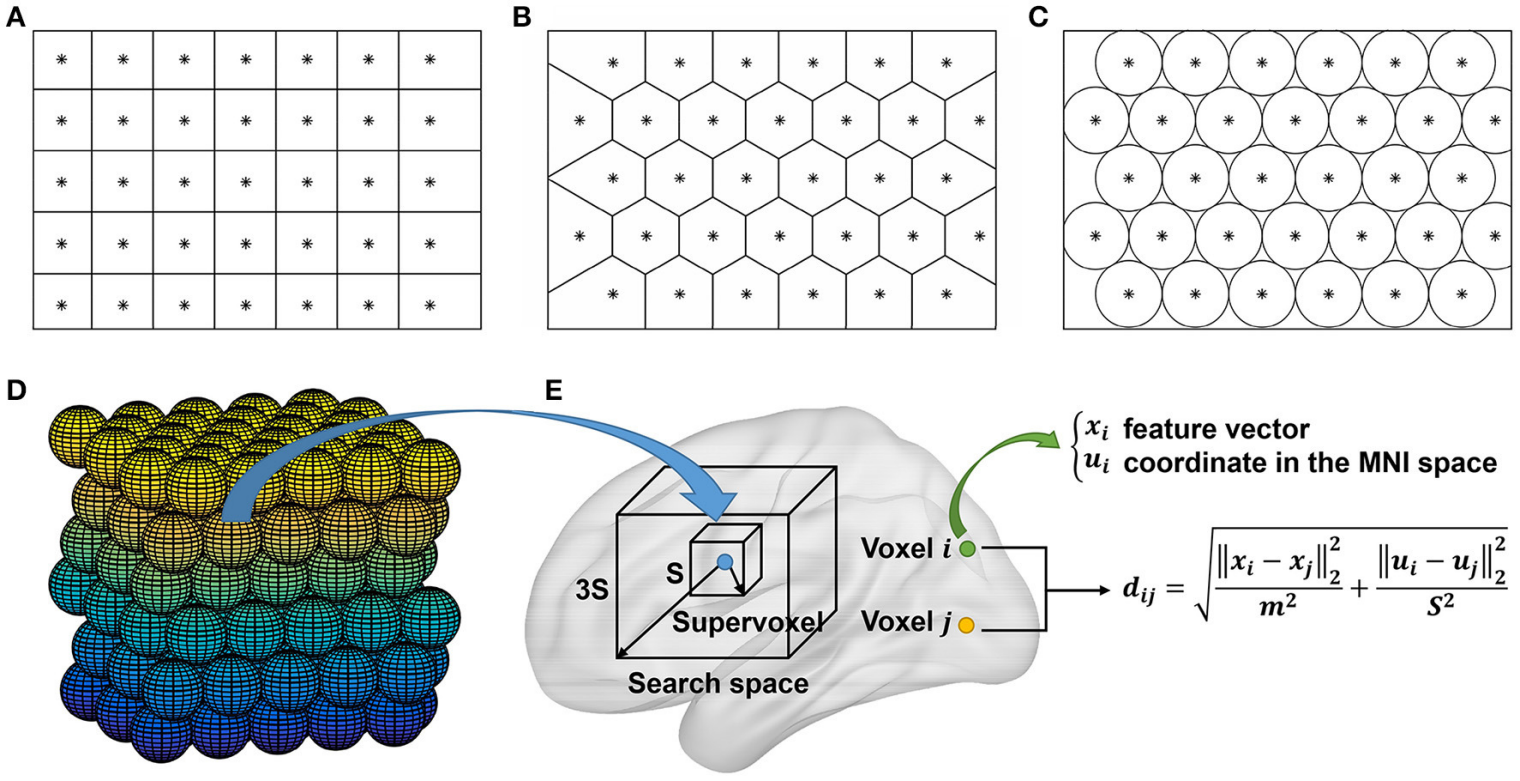
**(A–D)** Illustration of the initialization approaches in 2D and 3D spaces. The cluster centers are initialized by the centers of **(A)** square grids, **(B)** hexagonal grids, **(C)** circles, and **(D)** spheres, respectively. All of the units are stacked tightly in 2D or 3D space. The approach in **(B)** is an alternative to that in **(A)**, the approach in **(C)** is equivalent to that in **(B)**, and the approach in **(D)** is obtained by extending the approach in **(C)** from 2D to 3D space. **(E)** The searching step of the SLIC approach in whole brain parcellation. For each cluster, SLIC searches in the 3*S* × 3*S* × 3*S* region around its center to update the labels of all voxels in the search space. A unified distance is calculated between each voxel and the cluster center to judge whether the voxel should belong to the cluster. The unified distance is composed of the feature distance and the spatial distance, wherein the feature distance is calculated between the features of two voxels extracted by Ncut. Note that the supervoxel is displayed as a cube for simplicity, but it is unnecessary to be a cube.

Ideas for initialization of SLIC supervoxels could be borrowed from those of SLIC superpixels. There are two typical approaches for initialization of the SLIC superpixel method, i.e., initializing the cluster centers by the centers of square grids (Achanta et al., 2012) or hexagonal grids (http://www.peterkovesi.com/matlabfns/index.html#segmentation). Initialization by the second approach would result in a nominally 6-connected segmentation which is expected to facilitate subsequent postprocessing, thus being more preferred. Figures 2A,B show illustrations of the two approaches where an image is intended to be initialized with 35 cluster centers. The actual cluster number might be different from the initialized cluster number. Nevertheless, the difference is trivial; especially when the initialized cluster number is large and when the space to be parcellated is irregular as in brain parcellation.

The first approach is straightforward to be extended to 3D case (Lucchi et al., 2012). That is, we fill 3D space with cubes whose side length is $S=\sqrt[{3}]{N/K}$ and initialize the cluster centers by the cube centers. The extension of the second approach is indirect. Note that the same cluster centers as in Figure 2B could be obtained by filling 2D space with circles tightly and initializing the cluster centers by the circle centers, as shown in Figure 2C. Accordingly, the second approach could be extended to 3D case by filling 3D space with spheres tightly and initializing the cluster centers by the sphere centers, as shown in Figure 2D. We had tried the two approaches and found that their clustering performances were very similar. In this paper, we chose the second approach since spheres have higher symmetry than cubes. Figure 2E shows the searching step of the SLIC approach.

The variants of these initializations might introduce translations and rotations to the cluster centers, or even different spatial models rather than cubes and spheres. Additionally, the initialized clusters are not necessarily to be positioned periodically in the 3D space. A typical example is to move the initialized cluster centers to the locally optimal positions (Achanta et al., 2012; Blumensath et al., 2013). However, in practice, we found this approach would generate many empty clusters. Therefore, it was not applied.

The MSC and MKSC approaches also encounter initialization problems since there is a rotation matrix that should be initialized before iteration. Nevertheless, it was reported that these approaches are relatively robust to random initializations (Yu and Shi, 2003; Shen et al., 2013). Therefore, we initialized the rotation matrices randomly.

The SLIC approaches operate on a mixed space that is composed of the feature space and the Euclidean space. Therefore, we should define a unified distance by integrating the feature distance and the spatial distance between two voxels. Different from previous studies where the feature space is represented by the CIELAB color space (Achanta et al., 2012) or the image intensity space (Lucchi et al., 2012), the feature space in our study is composed of features extracted by Ncut. For the *i* th voxel in the gray matter mask, denote its corresponding feature computed by Ncut as *x*_*i*_ and denote its coordinate in the MNI space as *u*_*i*_. Then the unified distance between two voxels is defined as

$$\begin{array}{l} d_{ij}=\sqrt{\frac{{\left\|x_{i}-x_{j}\right\|}_{2}^{2}}{m^{2}}+\frac{{\left\|u_{i}-u_{j}\right\|}_{2}^{2}}{S^{2}},} \end{array}$$

where *m* and *S* are two tuning parameters. In this definition, the feature distance is normalized by *m* and the spatial distance is normalized by *S*. The parameter *m* is suggested to be fixed rather than to be dynamically estimated (Achanta et al., 2012). It should be carefully chosen because it adjusts the relative weights between the two distances and would lead to different clustering solutions. In our study, we normalized each feature vector to have zero mean and unit length before calculating the unified distance, and then we set *m* = 1 empirically. The parameter *S* is determined by the average cluster size that depends on cluster number but does not depend on cluster shape. Therefore, we set $S=\sqrt[{3}]{N/K}$ as in Lucchi et al. (2012).

#### Group level clustering

**Figure 3:**
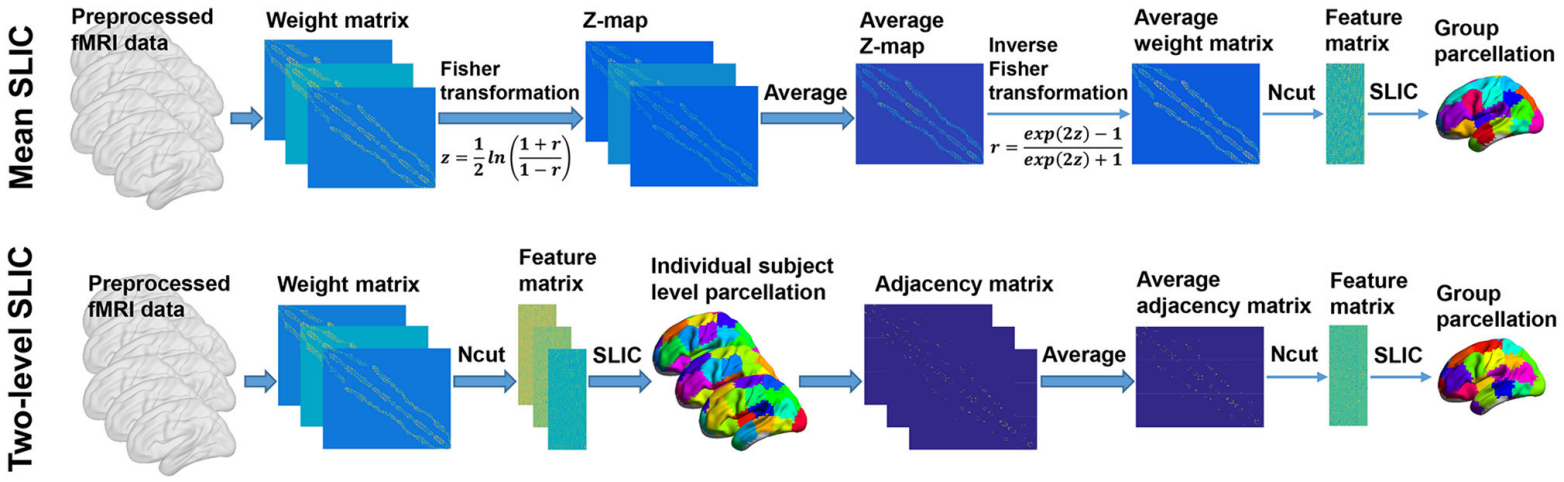
**The algorithm procedures of the mean SLIC and two-level SLIC approaches**. For both approaches, the calculations are operating at the individual subject level before the averaging step and at the group level after the averaging step. The parcellations are visualized with the BrainNet Viewer (http://www.nitrc.org/projects/bnv; Xia et al., 2013).

The group level clustering was achieved by adapting the two approaches in Craddock et al. (2012), yielding two approaches which were called mean SLIC approach and two-level SLIC approach. Figure 3 shows the algorithm procedures of the two approaches.

For the mean SLIC approach, we first generated a group-averaged weight matrix as in Yeo et al. (2011), Kahnt et al. (2012). Specifically, the individual weight matrices were converted into z-maps by Fisher's r-to-z transformation to increase the normality of the distribution of the correlations, then the z-maps were averaged across subjects, and finally an inverse Fisher's r-to-z transformation was applied to the averaged z-map to generate the group-averaged weight matrix. After obtaining the group-averaged weight matrix, we applied Ncut on it to extract features and then applied SLIC on the features to generate a group level parcellation.

For the two-level SLIC approach, we first applied Ncut and SLIC on the subject level weight matrix to generate a parcellation for each subject separately. Then for each parcellation, an adjacency matrix *A* was calculated by setting the element *a*_*ij*_ to be one if voxels *v*_*i*_ and *v*_*j*_ belonged to the same cluster and zero otherwise. The adjacency matrices were averaged across subjects to generate an averaged adjacency matrix that could be regarded as the second level weight matrix. Finally, we applied Ncut and SLIC on the averaged adjacency matrix to generate a group level parcellation. The idea of parcellating the brain in this two-level manner had been employed in studies such as van den Heuvel et al. (2008), Ryali et al. (2013, 2015) previously.

In our experiments, the competing approaches included the mean approach and the two-level approach in Craddock et al. (2012), and the MKSC approach in Shen et al. (2013). The two approaches from Craddock et al. (2012) are termed the mean MSC approach and the two-level MSC approach respectively in our study since the MSC algorithm is the veritable clustering algorithm in their parcellation procedures. The mean MSC approach was modified by employing the Fisher's r-to-z transformation, which slightly improved the reproducibility of the group parcellations. For the two-level MSC approach and the two-level SLIC approach, the cluster number at the individual subject level was set to be the same as at the group level. In the group level clustering, different sparsifying schemes could also be applied to the mean and second level weight matrices to make them sparser (van den Heuvel et al., 2008). Since these weight matrices were sparse already, for not introducing more tuning parameters, we did not perform any further processing on them.

#### The number of clusters

The selection of the optimal number of clusters remains to be an open problem in whole brain parcellation studies (Craddock et al., 2012; Moreno-Dominguez et al., 2014; Thirion et al., 2014). In our experiments, we initialized the cluster number by varying it from 50 to 1000 with a step of 50 in order to make a direct comparison with previous studies (Craddock et al., 2012; Blumensath et al., 2013). From the viewpoint of neurobiological interpretability, it is generally agreed that the two sides of this range represent two extremes in which cases the cluster number is either too small or too large, and the appropriate compromise lies inside this range (Fan et al., 2016; Glasser et al., 2016; Gordon et al., 2016).

### Evaluation criteria

The purpose of the whole brain parcellation is to generate atlases for human brain network analysis and RSFC studies. Three major criteria exist to evaluate the rationality of a brain atlas. That is, the clusters in a brain atlas should be spatially contiguous, functionally homogeneous, and reproducible (Craddock et al., 2012; Shen et al., 2013). Therefore, we evaluated the clustering performances of the parcellation approaches mainly by the three criteria.

The first criterion, i.e., spatial contiguity, is a necessary property of a “hard” parcellation (Smith et al., 2013). A hard parcellation approach generally requires that the clusters are non-overlapping, each cluster contains only one spatially contiguous region, and all voxels in the brain are assigned to their respective clusters. This paper focuses on hard parcellation approaches. The proposed SLIC approaches are based on Ncut and SLIC. The Ncut-based approaches are reported to have good spatial contiguity in related studies (Craddock et al., 2012; Shen et al., 2013). Besides that, SLIC stresses great emphasis on spatial structures by initializing an ideal geometric pattern, integrating the spatial distance into the unified distance, and searching in a local space. Therefore, we expect that it also would lead to good spatial contiguity. Few spatially discrete regions that belong to the same cluster might still exist in the generated atlases. Here, two regions are spatially discrete means that we could not find one voxel from each of them such that the two voxels are in the 26-connected neighborhoods of each other in 3D space. If so, we should identify these regions and assign a unique label to each of them. The increased cluster number is termed the spatial discontiguity index. A small number close to zero indicates good spatial contiguity.

For the second criterion, the clusters in a brain atlas are required to be functionally homogeneous, namely that the voxels in each cluster should have similar time courses (Zang et al., 2004). The homogeneity of the brain atlas could be defined by the average within-cluster similarity. Assume that there are *N* voxels being parcellated into *K* clusters; the voxel number in the *k* th cluster *C*_*k*_ is *n*_*k*_, *k* = 1, 2, …, *K*; the similarity between voxels *i* and *j* is *s*_*ij*_ which in our study equals the Pearson correlation coefficient *corr*(*v*_*i*_, *v*_*j*_), *i, j* = 1, 2, …, *N*. The average similarity within the *k* th cluster is

$$a(k)=\frac{1}{n_{k}(n_{k}-1)}\sum_{i,j\in C_{k},\text{ i }\neq \;j}s_{ij}.$$

Then the functional homogeneity of the brain atlas is calculated by averaging the similarity across clusters

$$Homogeneity=\frac{1}{K}\sum_{k=1}^{K}a(k).$$

Clusters that contain only one voxel are omitted in calculation. To avoid circular analysis, we trained an atlas and calculated its homogeneity on two separate groups of data. The higher the homogeneity results the better.

For the third criterion, the reproducibility of a clustering algorithm could be evaluated by Dice coefficient (Dice, 1945), measuring the similarity between two atlases (Blumensath et al., 2013; Shen et al., 2013) or between two adjacency matrices derived from the two atlases (Craddock et al., 2012). In the former scheme, one should iteratively match the regions between two atlases and then calculate a weighted average Dice coefficient across all matched pairs. This scheme is prone to suffer from instabilities since the numbers of regions of two atlases might be different, region shapes and voxel numbers of two matching regions might be different, and the matching order might change. The alternative scheme, that is, measuring Dice coefficient between two adjacency matrices could escape from these problems, thus being adopted in this study. For two adjacency matrices *A* and *B* derived from two atlases, the Dice coefficient is

$$\begin{array}{l} Dice=\frac{2|A\cap B|}{\left|A|+|B\right|}, \end{array}$$

where |·| denotes the number of ones in an adjacency matrix, *A* ∩ *B* denotes the union of the two adjacency matrices. To be specific, define *C* = *A* ∩ *B*, then *C*_*ij*_ is set to be one if and only if *A*_*ij*_ and *B*_*ij*_ are both ones, and *C*_*ij*_ is set to be zero otherwise. When evaluating reproducibility, the two atlases being compared should be generated independently in order to avoid bias. This was achieved by separating the subjects into two sets and training an atlas on each of them. In our study, both the group-to-group reproducibility and the group-to-subject reproducibility were investigated. Since we aim to obtain brain atlases that would be widely applicable, high reproducibility results are more preferred.

## Results

In the experiments, we combined the three sparsifying schemes, i.e., SS1, SS2, and SS3, with the five parcellation approaches, i.e., the mean MSC, two-level MSC, MKSC, mean SLIC, and two-level SLIC approaches, to yield fifteen kinds of parcellations and then compared their clustering performances under different evaluation metrics.

**Figure 4:**
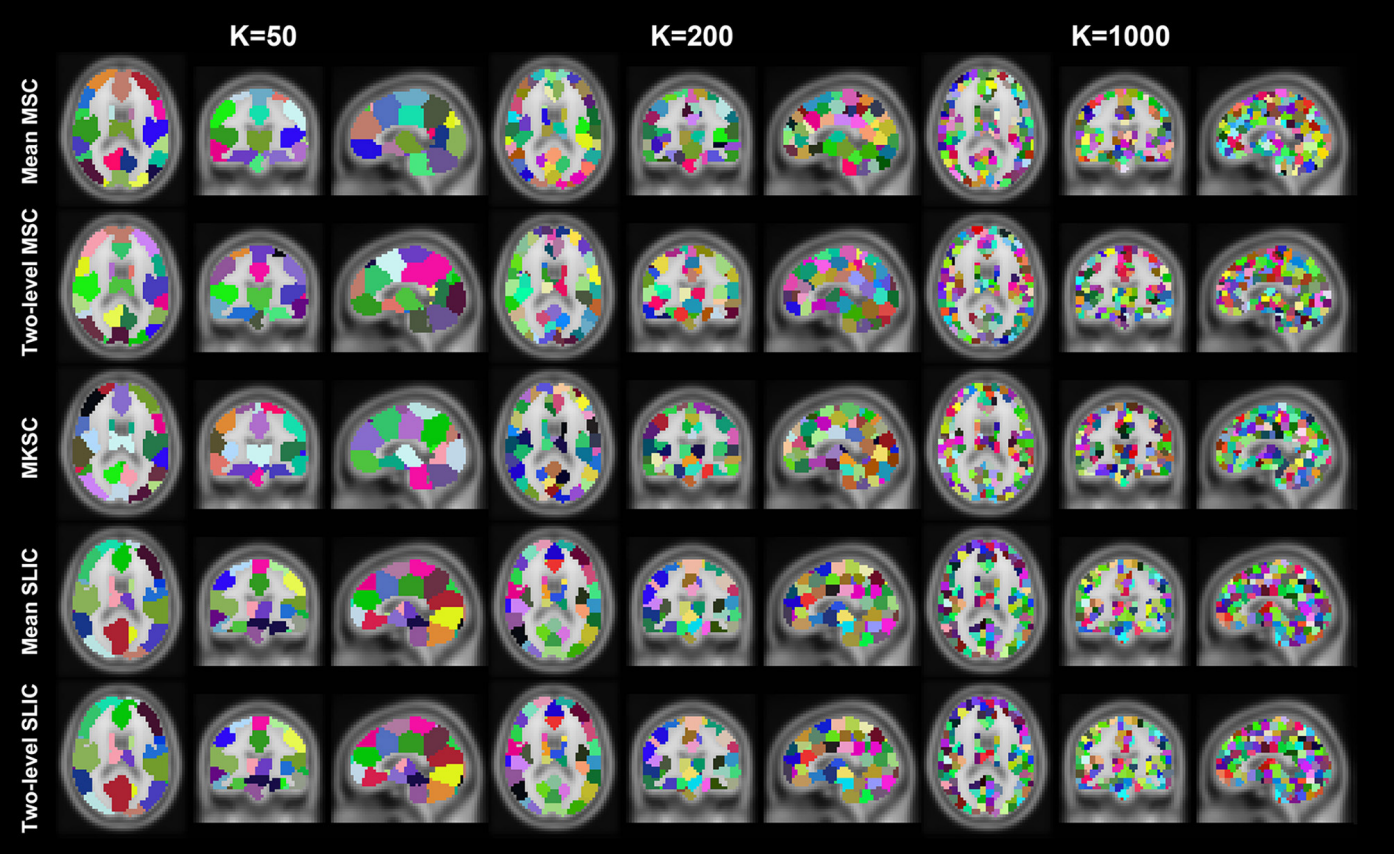
**Illustration of the atlases computed by the five parcellation approaches with SS1 when the cluster number is set to be 50, 200, and 1000**. Each atlas is represented by its three orthogonal cross sections. The colormap for each atlas is randomly generated, and each color represents a cluster.

**Figure 5:**
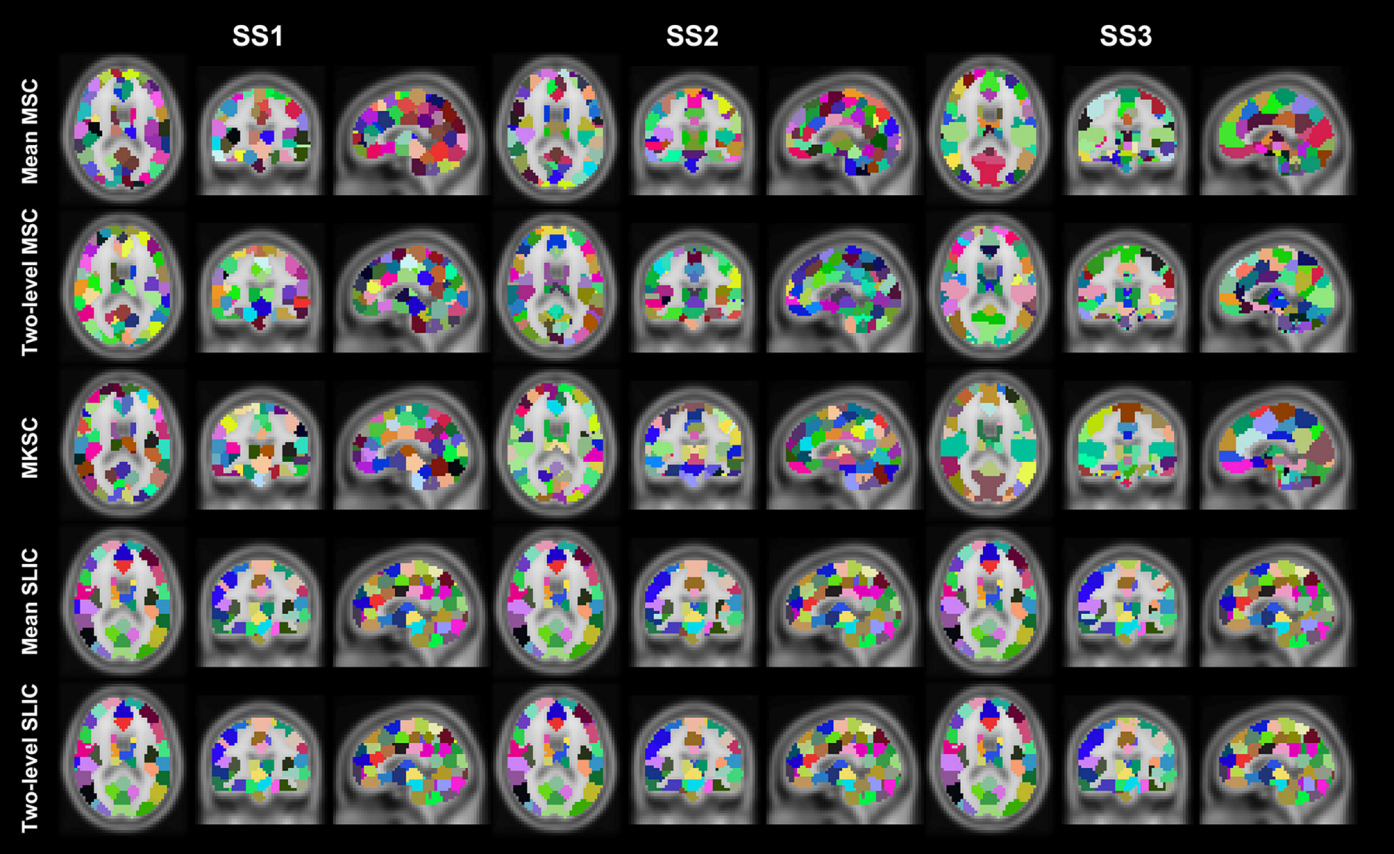
**Illustration of the atlases computed by the five parcellation approaches and the three sparsifying schemes when the initialized cluster number is 200**. Each atlas is represented by its three orthogonal cross sections. The colormap for each atlas is randomly generated, and each color represents a cluster.

Resting-state fMRI data from 40 subjects were utilized in the experiments. The 40 subjects were randomly separated into two groups, 20 subjects in each group. This procedure was repeated 10 times, resulting in 10 pairs of groups. For each parcellation approach, each sparsifying scheme, each cluster number, and each pair of groups, we obtained two group level parcellations based on the two groups of data. Figures 4, 5 show the illustrations of the atlases with different parcellation approaches, different sparsifying schemes, and different cluster numbers. These methods were applied on a random group of subjects. Supplementary Figures 1, 2 show the same atlases by mapping them onto the inflated cortical surface. From the figures, SS1 and SS2 tended to generate clusters with similar sizes and regular shapes while SS3 behaved differently.

### Actual cluster number

**Figure 6:**
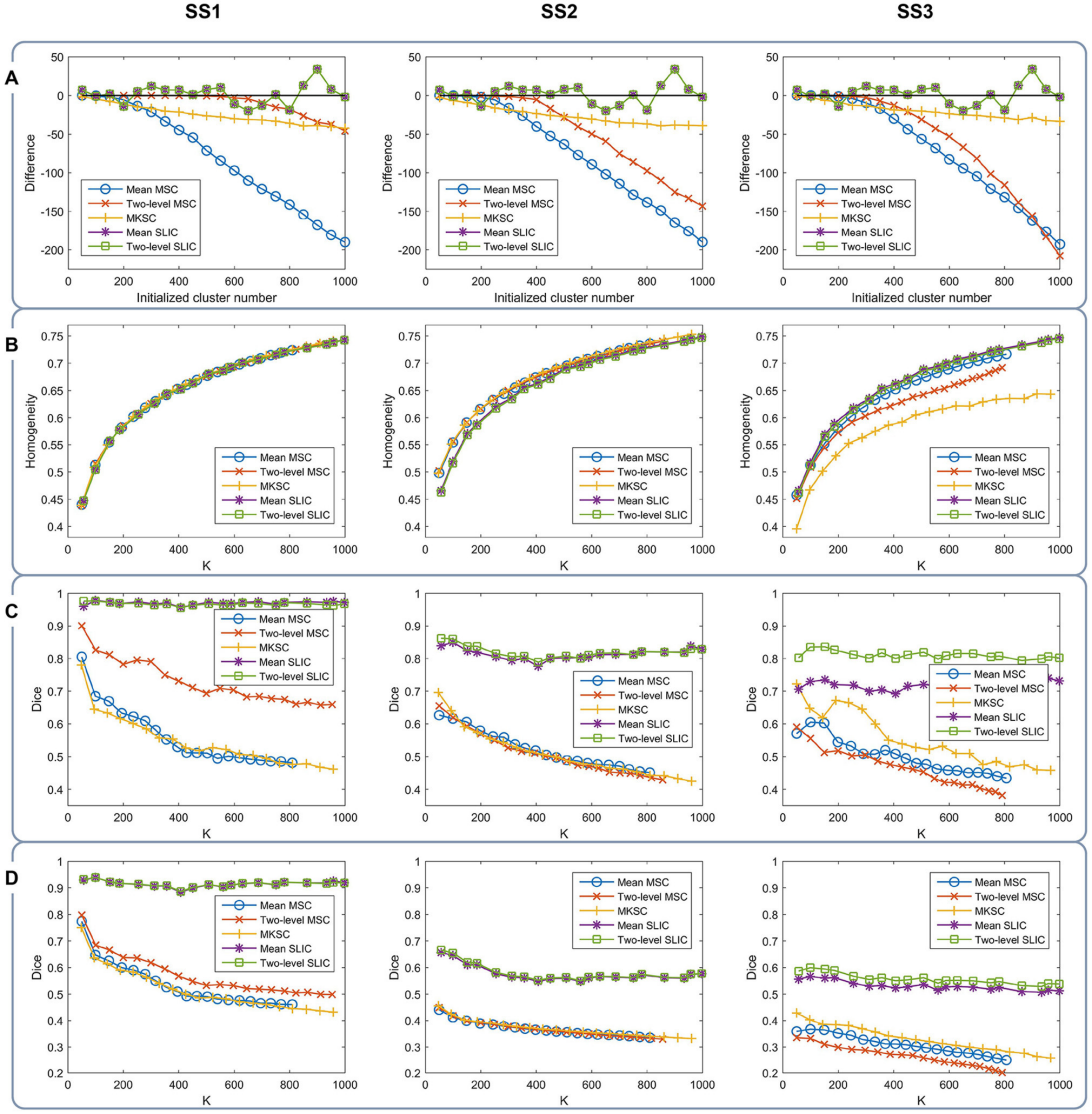
**The results of different evaluation metrics of the five parcellation approaches and the three sparsifying schemes**. The four rows correspond to **(A)** the difference between the actual cluster number and the initialized cluster number, **(B)** the functional homogeneity, **(C)** the group-to-group reproducibility, and **(D)** the group-to-subject reproducibility in order. The three columns correspond to SS1, SS2, and SS3 in order.

With a desired parcellation approach, the actual cluster number of the resultant atlases should be the same as the initialized cluster number. However, they are usually different due to the intrinsic properties of the parcellation approaches. Therefore, the actual cluster number should be counted when evaluating a parcellation approach. By subtracting the initialized cluster number from the average actual cluster number, we could obtain their differences. Figure 6A shows the average results of the differences for the five parcellation approaches and the three sparsifying schemes.

For the mean MSC, two-level MSC, and MKSC approaches, the differences were negative because these approaches generated many empty clusters (Craddock et al., 2012). Another property of the three approaches was that the absolute values of their differences were increasing with the initialized cluster number. For the mean SLIC and two-level SLIC approaches, the differences were fluctuating near zero, and the magnitudes were smaller than the other three approaches in most cases. Therefore, the SLIC approaches outperformed the other three approaches in approximating the initialized cluster number. The three sparsifying schemes had few influences on the results except when they were combined with the two-level MSC approach.

### Spatial contiguity

To calculate the spatial discontiguity index, we separated the spatially discrete regions belonging to the same cluster and counted the increased cluster number for each brain atlas. The results were averaged across all 400 atlases for each parcellation approach and each sparsifying scheme, as listed in Table 2. The average increased cluster numbers of SS1 were much smaller than the corresponding results of SS2 and SS3, especially for the mean MSC, two-level MSC, and MKSC approaches. It supports the conclusion in Craddock et al. (2012) which stated that it is necessary to apply spatial constraint to the weight matrix in order to enforce spatial contiguity to a parcellation.

**Table 2:** **The spatial discontiguity indices of the five parcellation approaches and the three sparsifying schemes**.

	**SS1**	**SS2**	**SS3**
Mean MSC	0.05	8.46	22.09
Two-level MSC	2.14	11.74	374.04
MKSC	0.53	5.71	206.31
Mean SLIC	1.00	1.23	1.48
Two-level SLIC	1.06	1.43	2.25

Both SS2 and SS3 did not impose spatial constraint on the weight matrix, but SS2 performed much better than SS3. To explain the result, we examined the subject level weight matrices generated by the three sparsifying schemes. Figure 1A shows an illustration of the three kinds of weight matrices. Most (67.50 ± 1.59%) of the non-zero elements in the weight matrices generated by SS2 fell into the spatial constraint while the proportion corresponding to SS3 was much smaller (42.88 ± 4.97%). For SS2, the coincidence is not surprising because voxels close in the Euclidean space tend to have high functional connectivity, and then the largest values in each row and each column tend to fall into the spatial constraint. In other words, though SS2 does not employ spatial constraint explicitly, it has a substantial relationship with spatial constraint. This is the reason why Shen et al. (2013) declared to obtain spatially contiguous parcellations without applying spatial constraint. For SS3, since the threshold is set globally to the weight matrix, much more non-zero elements fall outside the spatial constraint. Consequently, the parcellations of SS3 contain much more spatially discrete regions. For SS1, there are very few (0.06 ± 0.04%) negative elements and very few (3.50 ± 1.16%) elements smaller than 0.5 within the spatial constraint. With the negative and weak weights, the corresponding parcellations still exhibit good spatial contiguity.

When comparing the spatial contiguity across the five parcellation approaches, the mean SLIC and two-level SLIC approaches greatly outperformed the other three approaches when SS2 and SS3 were applied. It indicates that the SLIC approaches intrinsically enforce spatial contiguity and the other three approaches do not possess this ability. The reason is that SLIC incorporates spatial structures in the clustering procedure, while the MSC and MKSC algorithms do not.

In conclusion, the spatial contiguity largely depends on the spatial structures, which could be introduced by the weight matrix or by the SLIC algorithm. Only with appropriate spatial structures, the resultant parcellations could have satisfying spatial contiguity.

### Functional homogeneity

Then we investigated the homogeneity of the parcellations. For each parcellation approach, each sparsifying scheme, and each cluster number, there were 10 pairs of atlases. For each atlas, homogeneity was calculated based on the atlas and the resting-state fMRI data of the remaining 20 subjects that did not participate in generating the atlas. Then the homogeneity results were averaged across the 20 atlases. Figure 6B shows the averaged homogeneity results. Note that homogeneity was plotted against the average actual cluster number rather than the initialized cluster number. It could be observed that homogeneity generally increased with increasing cluster number, which is consistent with Craddock et al. (2012), Gordon et al. (2016). In addition, the homogeneity of a parcellation was influenced both by the parcellation approach and the sparsifying scheme.

**Figure 7:**
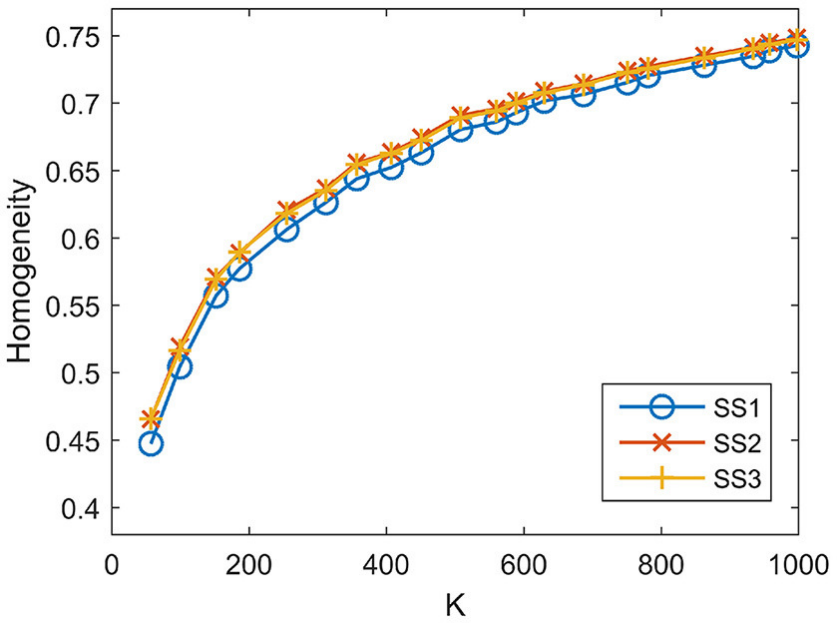
**Homogeneity results of the three sparsifying schemes when the parcellation approach is fixed to be the mean SLIC approach**.

For SS1, the homogeneity results of the five approaches fitted to the same curve. That is to say, when SS1 is employed, homogeneity has little relevance to the parcellation approach. It is similar to the results in Craddock et al. (2012) where the modified silhouette width turns out to be very close for various clustering methods. The homogeneity results of the mean MSC, two-level MSC, and MKSC approaches fitted to the same curve for SS2, but were quite different for SS3. The two SLIC approaches obtained similar homogeneity results with any of the three sparsifying schemes. By putting the homogeneity results of the mean SLIC approach for the three sparsifying schemes together, we obtained Figure 7. The results of SS2 and SS3 were close, both of which were slightly better than the results of SS1. Based on this comparison, we infer that the best homogeneity results are obtained by the mean MSC, two-level MSC, and MKSC approaches with SS2; the second best homogeneity results are obtained by the mean SLIC and two-level SLIC approaches with SS2 or SS3.

**Figure 8:**
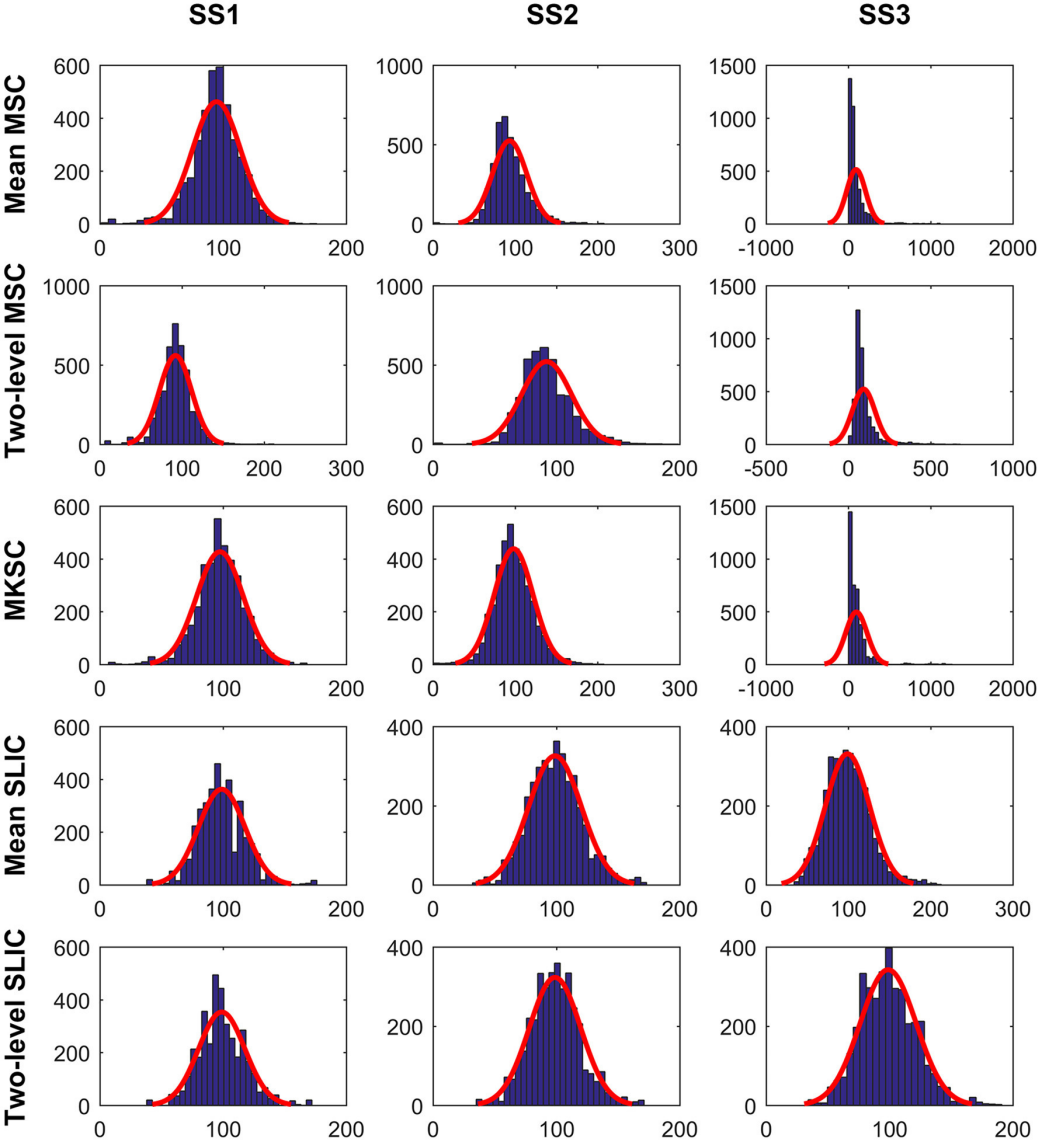
**The histograms of the cluster sizes for the five parcellation approaches and the three sparsifying schemes when the initialized cluster number is 200**. A normal distribution is fitted to each histogram in red.

The cluster homogeneity tends to decrease with increasing cluster size (Gordon et al., 2016). This could be derived from the fact that the homogeneity of a brain atlas tends to increase with increasing cluster number since increasing cluster number indicates decreasing cluster size. With the consideration, we tried to explain the homogeneity results from the distribution of cluster sizes. Figure 8 shows the histograms of the cluster sizes for different parcellation approaches and different sparsifying schemes when the initialized cluster number is 200. Except for the mean MSC, two-level MSC, and MKSC approaches with SS3, the histograms of the other methods all fitted well to normal distributions with similar parameters. It indicates that the clusters in the resultant parcellations have comparable sizes. The homogeneity results corresponding to the three exceptions were the worst among all methods. Therefore, the homogeneity of a brain atlas is influenced by the distribution of its cluster sizes. The distribution of cluster sizes depends on spatial structures in the sense that SS1 and SS2 are related with spatial constraint and the SLIC algorithm introduces spatial structures in the clustering procedure. Therefore, the homogeneity results could be explained by the spatial structures to a certain degree.

The calculation of homogeneity involves not only the parcellation, but also the functional connectivity of the fMRI data that does not participate in generating the parcellation. Some related factors that influence the fMRI data are the partial volume effects and susceptibility artifacts due to low image resolution (Craddock et al., 2012), and the smoothing effects in the preprocessing procedure. These factors might diminish the differences of the homogeneity results between different conditions.

### Group-to-group reproducibility

The group-to-group reproducibility was evaluated by the Dice coefficient (Dice, 1945; Craddock et al., 2012). Specifically, we first calculated a Dice coefficient for each pair of independently generated atlases and then averaged the Dice coefficients across the 10 pairs. Figure 6C shows the average Dice coefficient for each parcellation approach, each sparsifying scheme, and each cluster number. Similarly, it was plotted against the average actual cluster number.

The SLIC approaches greatly outperformed the other three approaches regardless of the sparsifying scheme. The reason is likely to be that SLIC introduces spatial structures in the clustering procedure while MSC and MKSC do not. It demonstrates that the atlases generated by the SLIC approaches generalize well between different groups of data. Comparing between the two SLIC approaches, two-level SLIC outperformed mean SLIC when SS3 was employed. With either of the other two sparsifying schemes, the two SLIC approaches obtained very close results. For SS1, two-level MSC outperformed mean MSC, which is consistent with Craddock et al. (2012). However, for SS2 and SS3, mean MSC generally outperformed two-level MSC. Therefore, which one is better in the two MSC approaches is largely determined by the sparsifying scheme. A similar conclusion could be obtained when MKSC is added for comparison since MKSC outperformed the two MSC approaches for SS3 but did not show any advantages over them for SS1 and SS2.

The Dice coefficients of the mean MSC, two-level MSC, and MKSC approaches were generally decreasing with increasing cluster number, which is consistent with Craddock et al. (2012), Blumensath et al. (2013), Shen et al. (2013). In Blumensath et al. (2013), the Dice similarity increased with increasing cluster number for most approaches except the MSC approach. The causes are suggested to be the spatial constraint and the restriction on cluster size. This explanation is applicable to SS1 and SS2 since the two sparsifying schemes are closely related to the spatial constraint and their resultant clusters are prone to have comparable sizes and shapes, as shown in Figures 4, 5, 8. For the two SLIC approaches, the Dice coefficients were relatively stable with different cluster numbers. Their trends are not only influenced by the spatial constraint, but also influenced by the additional spatial structures introduced by SLIC.

By comparing the Dice coefficients across different sparsifying schemes for each parcellation approach, we found that in most cases, the results of SS2 were worse than the results of SS1, and the results of SS3 were even worse. Considering the relationships between their weight matrices and the spatial constraint, we could infer that the Dice coefficients are positively influenced by the spatial constraint.

As discussed above, the Dice coefficients could be explained by spatial structures from three different kinds of viewpoints. It indicates that the group-to-group reproducibility relies largely on spatial structures.

### Group-to-subject reproducibility

To assess the ability of a group level parcellation to generalize to an individual subject level parcellation, we calculated the Dice coefficient between the group level atlas generated by a group of subjects and the individual subject level atlases generated by the subjects in the complementary group. The results were then averaged across subjects and groups. Note that the mean SLIC and two-level SLIC approaches reduced to the same approach when parcellating an individual subject. Similarly, the mean MSC, two-level MSC, and MKSC approaches reduced to the same approach when parcellating an individual subject. Figure 6D shows the results of group-to-subject reproducibility. The Dice coefficients decreased comparing to the corresponding results in Figure 6C. Nevertheless, most of conclusions from Figure 6C were applicable to the results in Figure 6D. Most importantly, the two SLIC approaches still greatly outperformed the other three approaches regardless of the sparsifying scheme. The results demonstrate the consistency between the group-to-group reproducibility and the group-to-subject reproducibility.

In conclusion, the proposed SLIC approaches could obtain relatively good overall clustering performances with different evaluation metrics. The SLIC approaches combine Ncut and SLIC. Ncut effectively captures the spatial structures from weight matrices, and leads to good results in terms of spatial contiguity and functional homogeneity consequently. On the other hand, SLIC greatly improves reproducibility, which indicates better generalization ability across different groups of data. Then by combining Ncut and SLIC, the resultant parcellations could obtain good results in all of the three evaluation criteria, which fulfills our original purpose well.

### Influences of confounding factors

Considering that many confounding factors might affect the parcellation results, it is valuable to investigate their influences, as stated below. Another two sparsifying schemes by which we tried to match the sparsifying schemes in Craddock et al. (2012) and Shen et al. (2013) were also investigated. See the Supplementary Materials. In these experiments, we only randomly separated the subjects into two groups twice in order to save computational time. Table 3 lists the results of different evaluation metrics of all experiments in order to make a full comparison. They were calculated by averaging the results across different cluster numbers for each parcellation method.

**Table 3:** **The average results of the evaluation metrics for different parcellation methods**.

	**Correlation**					**Correlation (GSR)**			**Correlation (OC)**			**Gaussian**			**One**		
	**SS1**	**SS2**	**SS3**	**SS4**	**SS5**	**SS1**	**SS2**	**SS3**	**SS1**	**SS2**	**SS3**	**SS1**	**SS2**	**SS3**	**SS1**	**SS2**	**SS3**
**DIFFERENCE**																	
Mean MSC	81.25	76.87	72.11	81.30	79.36	81.64	75.89	72.95	/	/	/	81.49	76.01	72.83	84.80	100.61	80.08
Two-level MSC	9.94	48.84	61.18	13.59	54.49	10.11	49.58	60.30	17.91	55.24	63.73	9.56	49.61	62.36	~	49.65	50.75
MKSC	25.05	26.02	19.95	26.90	22.45	25.35	26.15	19.20	/	/	/	24.95	25.64	18.69	~	25.04	8.84
Mean SLIC	9.75	9.75	9.75	9.75	9.75	9.75	9.75	9.75	/	/	/	9.75	9.75	9.75	9.75	9.75	9.75
Two-level SLIC	9.75	9.75	9.75	9.75	9.75	9.75	9.75	9.75	9.75	9.75	9.75	9.75	9.75	9.75	~	9.75	9.75
**SPATIAL DISCONTIGUITY INDEX**																	
Mean MSC	0.05	8.46	22.09	0.03	17.08	0.04	8.79	21.84	/	/	/	0.06	8.14	21.66	0.15	79.23	104.96
Two-level MSC	2.14	11.74	374.04	1.21	24.54	1.94	11.85	386.20	1.50	5.30	858.44	2.56	11.78	353.43	~	12.30	504.04
MKSC	0.53	5.71	206.31	0.53	12.33	0.69	5.79	208.08	/	/	/	0.51	5.86	210.21	~	6.15	242.54
Mean SLIC	1.00	1.23	1.48	0.95	1.40	0.95	1.20	1.44	/	/	/	1.00	1.14	1.39	0.90	1.69	1.76
Two-level SLIC	1.06	1.43	2.25	1.00	1.61	1.11	1.45	2.20	1.64	0.71	1.28	1.21	1.45	2.03	~	1.43	2.33
**FUNCTIONAL HOMOGENEITY**																	
Mean MSC	0.6442	0.6677	0.6431	0.6445	0.6684	0.5427	0.5726	0.5569	/	/	/	0.6443	0.6680	0.6431	0.6449	0.6681	0.6350
Two-level MSC	0.6558	0.6726	0.6225	0.6551	0.6732	0.5576	0.5775	0.5261	0.6523	0.6666	0.6331	0.6559	0.6726	0.6230	~	0.6731	0.6116
MKSC	0.6533	0.6761	0.5828	0.6529	0.6781	0.5542	0.5817	0.4945	/	/	/	0.6533	0.6758	0.5817	~	0.6765	0.5880
Mean SLIC	0.6562	0.6663	0.6650	0.6566	0.6668	0.5575	0.5702	0.5694	/	/	/	0.6562	0.6663	0.6650	0.6594	0.6653	0.6634
Two-level SLIC	0.6561	0.6639	0.6619	0.6565	0.6641	0.5574	0.5673	0.5653	0.6486	0.6593	0.6586	0.6560	0.6639	0.6618	~	0.6638	0.6616
**GROUP-TO-GROUP REPRODUCIBILITY**																	
Mean MSC	0.5570	0.5186	0.4974	0.5553	0.5051	0.5579	0.5235	0.4969	/	/	/	0.5630	0.5233	0.4948	/	0.4366	0.4312
Two-level MSC	0.7280	0.5045	0.4599	0.7002	0.4832	0.7273	0.5036	0.4569	0.5969	0.4894	0.3930	0.7357	0.5043	0.4590	/	0.5036	0.4682
MKSC	0.5465	0.5101	0.5539	0.5421	0.4941	0.5491	0.5106	0.5448	/	/	/	0.5461	0.5075	0.5501	/	0.5066	0.5214
Mean SLIC	0.9700	0.8135	0.7224	0.9640	0.8085	0.9702	0.8142	0.7214	/	/	/	0.9711	0.8144	0.7213	/	0.7712	0.6610
Two-level SLIC	0.9674	0.8194	0.8101	0.9578	0.8219	0.9677	0.8186	0.8119	0.7883	0.7502	0.7323	0.9687	0.8186	0.8103	/	0.8199	0.8191
**GROUP-TO-SUBJECT REPRODUCIBILITY**																	
Mean MSC	0.5318	0.3670	0.3065	0.5093	0.3385	0.5320	0.3672	0.3043	/	/	/	0.5396	0.3674	0.3056	/	0.3380	0.3057
Two-level MSC	0.5714	0.3654	0.2621	0.5434	0.3356	0.5712	0.3652	0.2596	0.4407	0.3567	0.2205	0.5799	0.3655	0.2627	/	0.3628	0.2825
MKSC	0.5180	0.3697	0.3304	0.5003	0.3405	0.5194	0.3697	0.3290	/	/	/	0.5222	0.3696	0.3319	/	0.3667	0.3312
Mean SLIC	0.9146	0.5767	0.5308	0.8758	0.5783	0.9146	0.5769	0.5306	/	/	/	0.9229	0.5767	0.5304	/	0.5683	0.5325
Two-level SLIC	0.9148	0.5804	0.5570	0.8776	0.5834	0.9149	0.5804	0.5576	0.4716	0.4777	0.4611	0.9227	0.5803	0.5574	/	0.5821	0.5762

*Difference, the average absolute value of the differences between the initialized cluster number and the average actual cluster numbers. SS4 and SS5 are defined in the Supplementary Materials. Slash (/), an indicator of vacancy in case where the evaluation metric does not exist. Tilde (~), an indicator of the same value as its upper one in case where different parcellation approaches are reduced to the same approach*.

#### Influences of global signal regression

**Figure 9:**
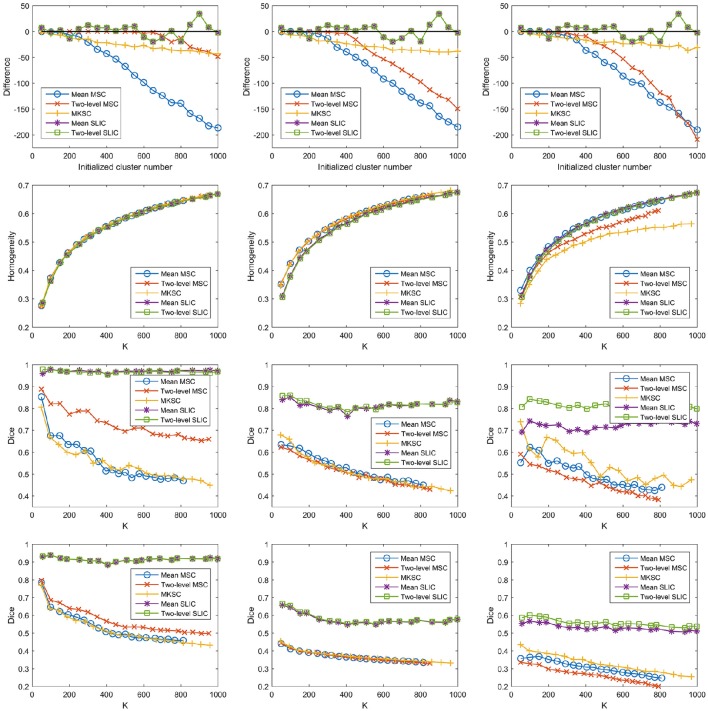
**The results of different evaluation metrics of the five parcellation approaches and the three sparsifying schemes when GSR is included in the preprocessing pipeline**. The four rows correspond to the difference between the actual cluster number and the initialized cluster number, the functional homogeneity, the group-to-group reproducibility, and the group-to-subject reproducibility in order. The three columns correspond to SS1, SS2, and SS3 in order.

In our study, we had applied a standard pipeline to preprocess the resting-state fMRI data. Changes in the preprocessing steps such as coregistration and spatial smoothing are likely to affect the final parcellations (Craddock et al., 2012; Shen et al., 2013), but it is difficult to investigate the influences of all potential changes. Possibly the most controversial step in the preprocessing pipeline is global signal regression (GSR; Yan et al., 2013). Therefore, we tried to investigate the influence of GSR on parcellations by incorporating GSR in the preprocessing pipeline. The three sparsifying schemes and the five parcellation approaches were applied likewise. The results of different evaluation metrics are shown in Figure 9 and Table 3. Most of the results were very close to the corresponding results on data without GSR except for that the functional homogeneity results became much lower. The reason might be that GSR leads to anticorrelations. Therefore, we chose to preprocess the resting-state fMRI data without GSR.

#### Influences of overclustering

**Figure 10:**
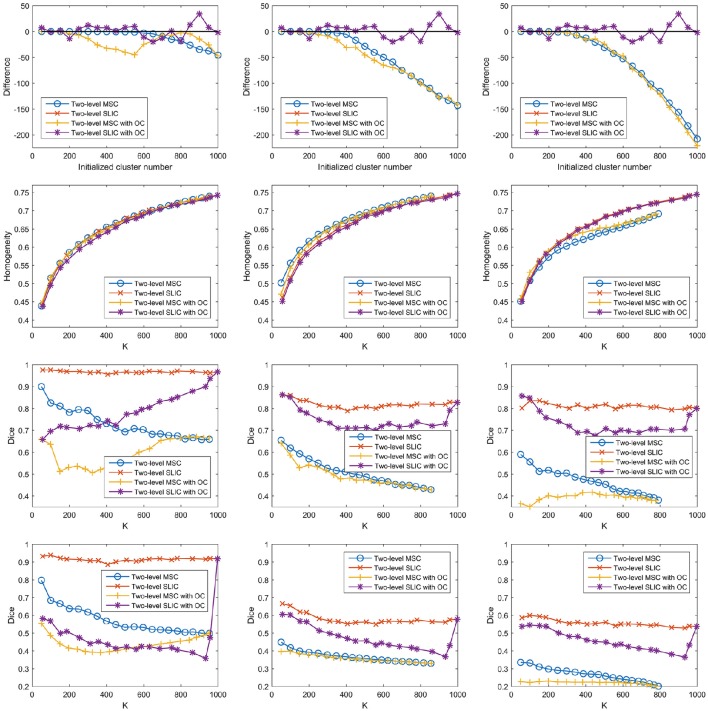
**The results of different evaluation metrics of the two-level approaches and the three sparsifying schemes**. The two-level approaches, i.e., the two-level MSC approach and the two-level SLIC approach, with and without overclustering (OC) are displayed for comparison. The four rows correspond to the difference between the actual cluster number and the initialized cluster number, the functional homogeneity, the group-to-group reproducibility, and the group-to-subject reproducibility in order. The three columns correspond to SS1, SS2, and SS3 in order.

For the two-level MSC approach and the two-level SLIC approach, the cluster number at the individual subject level was set to be the same as that at the group level by default. However, van den Heuvel et al. (2008) claimed that overclustering (OC) at the individual subject level would not change the nature of the group clustering results. We tried to validate this claim since it could reduce many computations. Specifically, we fixed the cluster number at the individual subject level to be 1000 and varied the cluster number at the group level from 50 to 1000 with a step of 50. With this setting, the two two-level approaches and different sparsifying schemes were employed to generate parcellations. The results of different evaluation metrics are shown in Figure 10 and Table 3, wherein the two-level approaches without overclustering are also included for comparison. On the one hand, with or without overclustering, the parcellations achieved very close results in terms of homogeneity. On the other hand, overclustering produced deteriorated reproducibility. Therefore, we did not apply overclustering by default. The influences of overclustering in van den Heuvel et al. (2008) and in our study are different. The reason might be that the cluster number in the individual subject level in van den Heuvel et al. (2008) is very small, i.e., from 15 to 45, but it is much larger in our study.

#### Influences of different weighting functions

**Figure 11:**
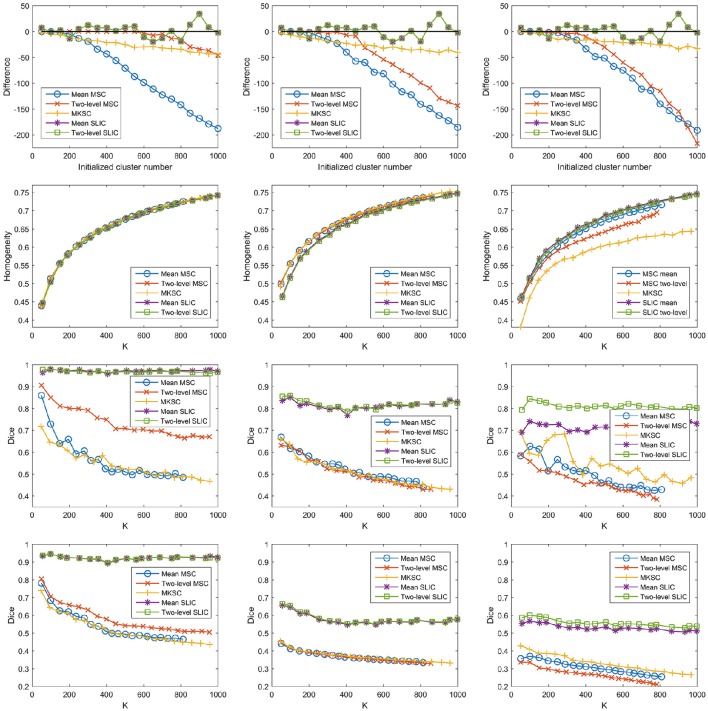
**The results of different evaluation metrics of the five parcellation approaches and the three sparsifying schemes when the weighting function is chosen to be the Gaussian kernel function**. The four rows correspond to the difference between the actual cluster number and the initialized cluster number, the functional homogeneity, the group-to-group reproducibility, and the group-to-subject reproducibility in order. The three columns correspond to SS1, SS2, and SS3 in order.

Then we proceeded to investigate the influences of different weighting functions. To do this, we changed the weighting function from the Pearson correlation coefficient to the Gaussian kernel function. The Gaussian kernel function with SS2 would generate the weight matrix in Shen et al. (2013). The three sparsifying schemes and the five parcellation approaches were then applied likewise. The results of different evaluation metrics are shown in Figure 11 and Table 3.

From those results, changing the weighting function from the Pearson correlation coefficient to the Gaussian kernel function would hardly affect the clustering performances.

A major reason for the result is that the clustering performance of a Ncut-based approach is stable with different weighting functions (Shi and Malik, 2000). To explain further, the Gaussian kernel function is positively related to the Pearson correlation coefficient by the following equation

$$\begin{array}{l} e^{-\frac{{\left\|v_{i}-v_{j}\right\|}_{2}^{2}}{\sigma^{2}}}=e^{-\frac{\left(1-corr\left(v_{i},v_{j}\right)\right)\times 2}{\sigma^{2}}}. \end{array}$$

However, when SS1 is employed, even if the weighting function is set to be a constant value one, the MSC approaches could obtain comparable results in terms of homogeneity and reproducibility (Craddock et al., 2012). It is known as the random parcellation, and it brings quite a lot of doubts to the rationality of the connectivity-based parcellations (Craddock et al., 2012; Blumensath et al., 2013; Shen et al., 2013; Gordon et al., 2016). To sum up, when SS1 is employed, different weighting functions such as the Pearson correlation coefficient, the Gaussian kernel function, and a constant function would not greatly affect the clustering performances.

**Figure 12:**
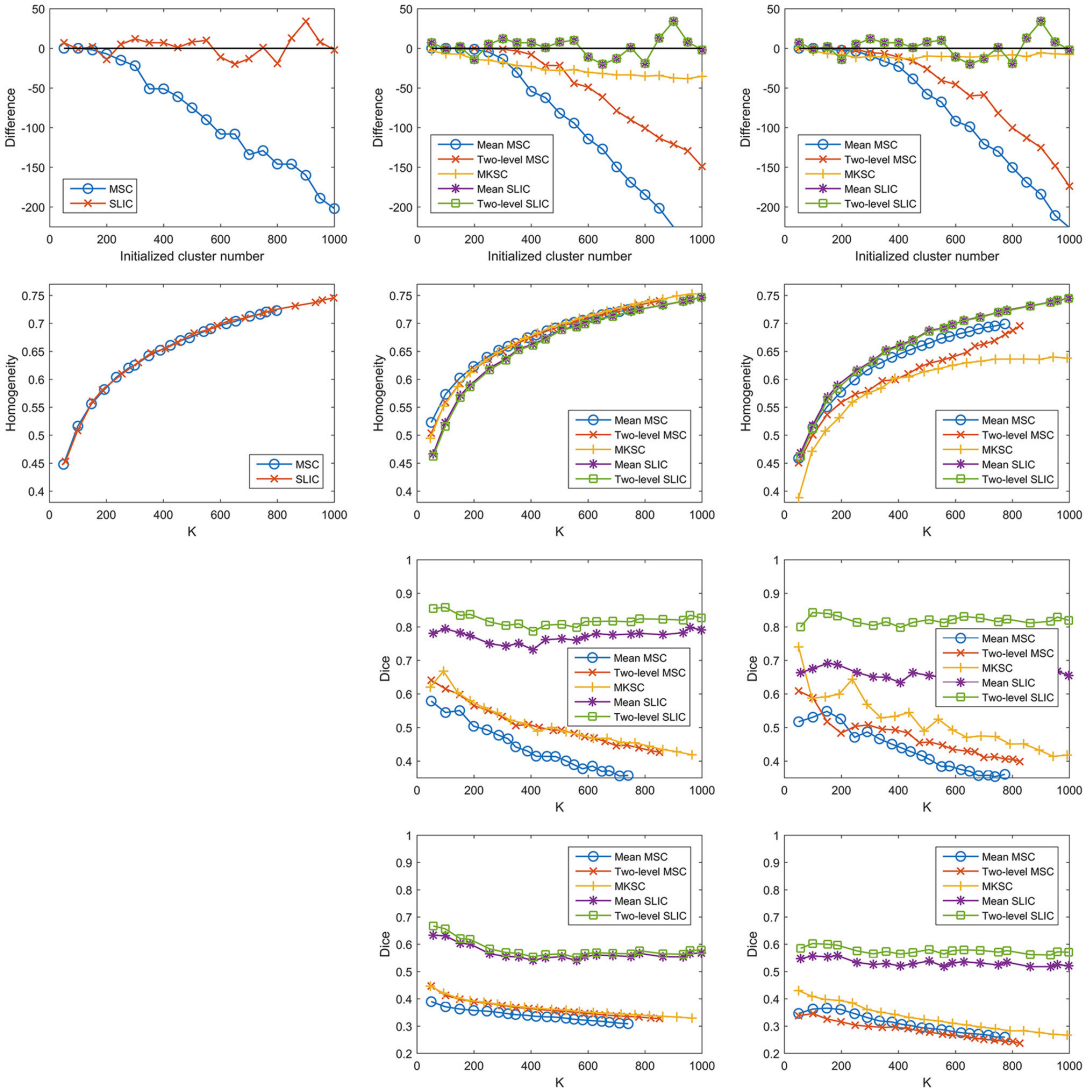
**The results of different evaluation metrics of the five parcellation approaches and the three sparsifying schemes when the weighting function is set to be a constant value one**. The four rows correspond to the difference between the actual cluster number and the initialized cluster number, the functional homogeneity, the group-to-group reproducibility, and the group-to-subject reproducibility in order. The three columns correspond to SS1, SS2, and SS3 in order.

Then it is interesting to investigate whether the other two sparsifying schemes also possess this property. To do this, we constructed trivial weight matrices (all reserved weights = 1) for the three sparsifying schemes. It was achieved by setting the non-zero elements in the three kinds of sparse weight matrices constructed by the Pearson correlation coefficient to be ones. This operation was only applied to the individual subject level weight matrix, but not to the second level weight matrix. The trivial weight matrices with SS1 was equal to the spatial constraint, which was identical for different subjects. In this case, the mean MSC, two-level MSC, and MKSC approaches reduced to the same approach that was denoted as the MSC approach. Meanwhile, the mean SLIC and two-level SLIC approaches reduced to the same approach that was denoted as the SLIC approach. For each of the two approaches, there was only one parcellation for each initialized cluster number. Therefore, no corresponding reproducibility was calculated. The results of different evaluation metrics on these trivial weight matrices corresponding to the three sparsifying schemes are shown in Figure 12 and Table 3.

The homogeneity results of the MSC approach on the spatial constraint were close to the homogeneity results of the mean MSC and two-level MSC approaches on the weight matrix with SS1, which is consistent with Craddock et al. (2012). For SS2 and SS3, the differences between the initialized cluster number and the actual cluster number of the mean MSC approach became lower, the reproducibility results of the mean MSC and mean SLIC approaches became lower, and the spatial discontiguity indices of all of the five approaches became larger. The remaining results were close to the corresponding results when the other two weighting functions were employed. To sum up, changing the weighting function from the Pearson correlation coefficient or the Gaussian kernel function to the constant function would lower some of the clustering performances, but most of the results would be similar.

A potential reason of this finding is that the reserved weights with the Pearson correlation coefficient are large, all close to ones, thus leading to similar clustering performances. It cannot fully explain the results, as shown in Figure 1C. Then the underlying reason might be the spatial structures incorporated in the parcellation approaches, which are invariant with different weighting functions. The spatial structures corresponding to different sparsifying schemes are different. This could explain why the results of the three sparsifying schemes have significant differences. In conclusion, all of the five parcellation approaches might rely heavily on spatial structures. This is the major limitation of the Ncut-based approaches.

### Average results

Table 3 concentrates the average results of different evaluation metrics. Since the actual cluster numbers of different parcellation methods are not the same, it is generally not appropriate to compare these methods by the overall results. An exception is that with the same parcellation approach and the same sparsifying scheme, setting the weighting function to be the Pearson correlation coefficient or the Gaussian kernel function would obtain very similar results in terms of the actual cluster number, though still not the same. Under the premise that the differences are negligible, we could compare the corresponding results. The results in this table demonstrate that the two weighting functions obtain very close clustering performances.

## Discussion

### Alternative algorithm procedures

The SLIC algorithm could be directly applied on the resting-state fMRI time series to perform parcellation, similar to the case when SLIC is applied to segment 3D images (Lucchi et al., 2012). By this way, the intensity information rather than the connectivity information is utilized. A study which built on this idea and focused on individual subject level parcellation had been previously presented in (Wang et al., 2016). By extending the idea, we could construct the mean approach and the two-level approach accordingly in order to generate group level parcellations. For the mean approach, the fMRI time series were concatenated or averaged across subjects. For the two-level approach, a two-level weight matrix was defined by the averaged adjacency matrix, and then SLIC was applied on the two-level weight matrix to perform parcellation. We had tried the two approaches and found that they tended to be worse than the connectivity-based parcellation approaches under the three evaluation metrics. The reason might be that connectivity information is much richer than intensity information.

Another category of parcellation approaches is to perform parcellation directly on the connectivity matrices. Namely, we skipped the step of extracting features from the connectivity matrices by Ncut. Except for this change, all of the other steps were the same as in the algorithm procedures in Figure 3. The major problem of this thought is that the dimensionality of the connectivity matrices is very high and it becomes much harder to compute in practice. In fact, Ncut is very important in the parcellation procedure not only because it could extract relevant features but also because it could effectively reduce data dimensionality.

### Factors that determine the parcellation

The factors that determine the functional connectivity-based parcellation could be concluded into five major aspects. The first aspect is the choice of the preprocessing pipeline of fMRI data. The impacts of different preprocessing steps such as coregistration and spatial smoothing had been discussed in Craddock et al. (2012), Shen et al. (2013). In our study, we had examined the influences of GSR and found that GSR led to worse homogeneity results. Therefore, we did not apply GSR in the preprocessing procedure by default. Generally, we made our parcellations reasonable by utilizing a standard preprocessing pipeline (Yan and Zang, 2010; Yan et al., 2013). The second aspect is the selection of the cluster number. Since there is no optimal choice as far as we know, we varied the cluster number from 50 to 1000 with a step of 50 to generate parcellations at multiple granularities. To achieve group level parcellations, we set the cluster numbers at the individual subject level and at the group level to be the same. From the results of functional homogeneity and reproducibility, we still could not find an optimal cluster number. Therefore, parcellations with different granularities might be applied to different studies in accordance with requirements. Note that they are not hierarchically consistent which is a property possessed only by parcellations generated from hierarchical clustering. The third aspect is the definition of the weight matrix. It includes the choice of the weighting function, the choice of the sparsifying scheme, the choice of parameters in the weighting function and the sparsifying scheme, whether to set the diagonal elements in the non-empty rows and columns to be zeros or ones, and whether to apply the sparsifying schemes to the mean and second level weight matrices or not. These factors were carefully investigated in our experiments. The fourth aspect is the parcellation approaches utilized. Attentions should be paid to the initialization of the SLIC supervoxels and the definition of the unified distance in SLIC. We initialized the supervoxel centers by the centers of tightly stacked spheres in 3D space. The unified distance is composed of the functional distance and spatial distance, and we set the parameter *m* in the unified distance to be one empirically. The fifth aspect is postprocessing. After obtaining the atlases by these parcellation approaches, we might separate the spatially distinct regions and merge some small clusters to make the atlases more reasonable (Achanta et al., 2012). However, from the results in Table 3 and Figure 8, there were only few spatially distinct regions and small clusters except when the mean MSC, two-level MSC, and MKSC approaches were combined with SS3. To avoid the influences of the postprocessing steps on functional homogeneity and reproducibility, we did not apply postprocessing on the atlases. Considerations on the above aspects guarantee the rationality of our parcellations.

### Limitation of the parcellation approaches

The SLIC approaches yield clusters with comparable shapes and sizes, which are unlikely to be the functional units (Glasser et al., 2016) in the brain. This is a common problem encountered by all of the Ncut-based approaches (Craddock et al., 2012; Shen et al., 2013) since they incorporate strong spatial structures represented by the spatial constraint in the clustering procedure. The spatial constraint is necessary in order to guarantee spatial contiguity for the MSC and MKSC approaches, as shown in the experiments. For the SLIC approaches, additional spatial structures are introduced by initializing an ideal geometric pattern, integrating the spatial distance into the unified distance, and searching in a local space. The additional spatial structures lead to improved reproducibility, but are likely to aggravate the aforementioned problem. Recently, Parisot et al. (2016) applied spectral clustering with spatial constraint on supervertices to perform whole brain parcellation and encountered the same problem. It is because spatial structures were introduced by the supervertex generation procedure and the spatial constraint.

Except for Ncut, a major category of whole brain parcellation approaches is based on hierarchical clustering (Blumensath et al., 2013; Moreno-Dominguez et al., 2014; Thirion et al., 2014; Parisot et al., 2016). In order to guarantee spatial contiguity of resultant clusters, spatial structures should be incorporated by only merging the neighboring regions. This rule also applies to K-means in whole brain parcellation (Parisot et al., 2016). For these clustering algorithms, the influences of the spatial structures on the resultant parcellations are yet to be investigated.

When subdividing a small ROI into a few clusters, the parcellation approach could be purely data-driven without incorporating spatial structures. For example, Kim et al. (2010) applied K-means to parcellate the medial frontal cortex into two subregions, Fan et al. (2016) applied spectral clustering to parcellate each of the 82 seed regions in the Desikan-Killiany atlas into 2–12 clusters. In these studies, no spatial constraint was applied. Since fMRI data are typically noisy and smooth, the generated clusters could have good spatial contiguity. However, these properties of fMRI data are not adequate to guarantee the spatial contiguity of whole brain parcellation approaches. Generally, spatial structures might be unnecessary when parcellating a small ROI, but are necessary when parcellating the whole brain. Therefore, it is urgent to find a proper way to tackle the limitations introduced by the spatial structures for whole brain parcellation approaches.

With the limitation, the proposed approaches are still well-suited to fulfill the original purpose of parcellating the brain into spatially contiguous, functionally homogeneous, and reproducible clusters. Consequently, they could find applications in various studies. For example, the atlases generated by the Ncut-based approaches had been successfully applied in tracking ongoing cognition (Gonzalez-Castillo et al., 2015), identifying individuals (Finn et al., 2015), measuring sustained attentional abilities (Rosenberg et al., 2016), etc. Therefore, we remain optimistic about our approaches and expect the generated atlases to facilitate related studies.

### Alternatives of the evaluation metrics

As far as we know, there is no gold standard to evaluate a functional connectivity-based parcellation. In other words, how to judge whether a parcellation is good or not still remains to be an open problem. A comprehensive review of different evaluation criteria and many related metrics could be found in Eickhoff et al. (2015). The review claimed that it is natural for the connectivity-based parcellation analysis to resort to exploratory statistics rather than inferential statistics, because it is difficult to formulate a null hypothesis for a parcellation to test against and consequently it is difficult to assess the statistical significance of the parcellation. The exploratory statistics include various cluster validity criteria, i.e., various evaluation criteria, and there are many different choices for each criterion. It is suggested that a parcellation should be assessed globally and synthetically using different cluster validity criteria, as done in this study. Some alternatives of the evaluation metrics employed in our experiments are discussed as follows.

Several alternative metrics concerning functional homogeneity are presented in related studies. In van den Heuvel et al. (2008), the Ncut cost is employed as an evaluation metric which measures inhomogeneity. In Craddock et al. (2012), a modified silhouette width (Rousseeuw, 1987) and an accuracy of representation are defined. The modified silhouette width considers not only within-cluster homogeneity but also between-cluster heterogeneity. The accuracy of representation intends to evaluate the ability of a brain atlas to represent the functional connectivity patterns at the voxel scale, and its result depends on the seed region chosen in prior. In Shen et al. (2013), an inhomogeneity metric is defined by the average within-cluster dissimilarity wherein the dissimilarity is defined by Euclidean distance. In Gordon et al. (2016), principal component analysis is applied on the whole brain connectivity patterns of each cluster, then the cluster homogeneity is defined by the percent of variance explained by the largest principal component, and finally the cluster homogeneity results are averaged to obtain the homogeneity of a brain atlas. These metrics are closely related to the functional homogeneity.

Alternative metrics for evaluating reproducibility include, but are not limited to, Jaccard index, Hausdorff distance, mutual information, variation of information, and Rand index (Shen et al., 2013; Thirion et al., 2014; Ryali et al., 2015). In our study, we focus on the Dice coefficient since it is widely used and very typical.

One way to extend the class of evaluation criteria is to measure the overlap between the clusters generated by connectivity-based parcellation approaches and the regions generated from task activation, myelin maps, cortical thickness, topography, or electrical cortical stimulation (Blumensath et al., 2013; Laumann et al., 2015; Wang et al., 2015; Parisot et al., 2016) etc. It provides external validity different from the aforementioned evaluation metrics. However, it is based on the assumption that neuroimaging data with different modalities should yield the similar parcellations, which has long been suspectable (Amunts et al., 2014; Eickhoff et al., 2015). Recently, Glasser et al. (2016) treated the spatially overlapping gradient ridges in at least two independent modalities as an areal border and found that the vast majority of areal borders satisfied this requirement. It provides a strong evidence for that assumption. Therefore, the rationality of the connectivity-based parcellations could be validated by comparing with multi-modal areal features. Nevertheless, it is difficult to quantify this evaluation criterion at whole brain level for parcellations with multiple granularities. This kind of evaluation is beyond the scope of our study.

Another independent evaluation criterion is the hierarchical consistency (Kahnt et al., 2012). That is, with an ideal hierarchical structure, the clusters in a fine parcellation should always stem from the clusters in a coarse parcellation. This parent-child congruency is perfectly guaranteed by hierarchical clustering (Blumensath et al., 2013; Moreno-Dominguez et al., 2014), but unlikely possessed by other kinds of clustering methods, especially when the cluster number is large. Hence, it is not discussed in this study.

### Limitation of the evaluation metrics

We used three different evaluation criteria, i.e., spatial contiguity, functional homogeneity, and reproducibility, to evaluate different classes of properties of a brain atlas. The evaluation metrics are majorly inherited from Craddock et al. (2012), Shen et al. (2013) in order to make a direct comparison with these studies. However, from the experiments, we find that these evaluation metrics have some inherent limitations.

The first criterion, i.e., spatial contiguity, is generally reasonable. It is unlikely that the clusters in a good parcellation contain many spatially discrete regions, though few ones are tolerable. There might be paired clusters across hemispheres due to the symmetrical property of the brain. However, they tend to be recognized as separate clusters due to long spatial distance. An exception is that some paired clusters might be joined together around the midline.

The second criterion, i.e., functional homogeneity, suffers from the problem that it could hardly differentiate various methods. As long as the clusters are spatially contiguous, e.g., when the sparsifying scheme is chosen to be SS1 or SS2, the homogeneity of a brain atlas is largely determined by the cluster number. Related metrics such as the modified silhouette width and the accuracy of representation also behaved like this in Craddock et al. (2012). Therefore, it is worthwhile to seek or design a metric that could evaluate homogeneity with higher discriminative power. Attentions should be paid to the factors that might influence functional connectivity since it is a key role other than the atlas in defining homogeneity.

The third criterion, i.e., reproducibility represented by the Dice coefficient, could distinguish different methods. However, a problem exists that we cannot definitely claim the higher the reproducibility the better. A high reproducibility result indicates more commonalities across subjects while a low reproducibility result indicates more idiosyncrasies from each subject. In this study, we aim to obtain group parcellations that would be widely applicable. Therefore, high reproducibility results are more preferred. From this viewpoint, the purposed approaches achieve good performances. Nevertheless, it should be cautious that reproducibility might rely on spatial structures heavily and render the influence of fMRI data very weak. This problem is difficult to be avoided in whole brain parcellation since it is necessary to incorporate spatial structures in order to guarantee the spatial contiguity of the generated atlases.

In conclusion, both spatial contiguity and reproducibility rely on spatial structures. When spatial structures are dominant in the parcellation procedure, the generated atlases might only weakly relate with fMRI data, as demonstrated by the random parcellation (Craddock et al., 2012). Measuring to what degree fMRI data is utilized in a parcellation is the task of homogeneity related metrics, but these metrics typically do not have enough power to distinguish different parcellation approaches. The limitations of the evaluation metrics complicate the studies of brain parcellation since the suitabilities of these metrics are yet to be evaluated. Moreover, parcellation approaches are usually designed to fit the purposes represented by these evaluation metrics. Then inappropriate evaluation metrics might increase the risk of overfitting or bias. It is therefore very urgent to seek or design some evaluation metrics that are necessary and sufficient to judge whether a parcellation is good or not. This is the prerequisite for a good brain parcellation approach and consequently good brain parcellations. As things stand, the most credible evaluation criterion is to compare a parcellation with multi-modal areal features manually by experienced neuroanatomists (Glasser et al., 2016).

### Future directions

For validation and application of the proposed approaches, it is meaningful to evaluate them on an independent dataset collected with a different scanner (Craddock et al., 2012; Gordon et al., 2016) and to apply the approaches to data with different modalities such as architecture, function, and topography (Blumensath et al., 2013; Finn et al., 2015; Wang et al., 2015). Integrating multi-modal neuroimaging data to carry out parcellation naturally guarantees the rationality of its findings (Glasser et al., 2016), thus also being worthwhile to explore. In addition, the inter-individual variability of the parcellations is worth studying since it is particularly important in applications such as development, aging, disease, and personalized medicine (Wang et al., 2015; Glasser et al., 2016).

For improving whole brain parcellation approaches, since existing approaches tend to rely heavily on spatial structures, it is necessary to find a way to weaken this dependence. In addition, the lack of a gold standard in evaluating a whole brain parcellation makes the efforts in algorithm design and evaluation very difficult, and thus it is very urgent to be dealt with.

## Conclusion

This paper presents two novel approaches, i.e., the mean SLIC approach and the two-level SLIC approach, to parcellate whole brain resting-state fMRI data into spatially contiguous, functionally homogeneous, and reproducible clusters. The proposed approaches integrated Ncut and SLIC. Specifically, Ncut was employed to extract features from connectivity matrices, and then SLIC was applied on the features to generate parcellations. Three existing Ncut-based parcellation approaches, i.e., the mean MSC, two-level MSC, and MKSC approaches, were compared with the proposed approaches in the study. The two SLIC approaches obtained relatively good overall performances. In terms of spatial contiguity, the SLIC approaches had evident advantages over the three competing approaches when the spatial constraint was not employed. In terms of functional homogeneity, the SLIC approaches obtained the second best results that were just slightly lower than the best ones. In terms of reproducibility, the SLIC approaches greatly outperformed the three competing approaches, both in the group-to-group aspect and the group-to-subject aspect. In addition, we had investigated the influences of GSR, overclustering, different weighting functions, and different sparsifying schemes on clustering performances. The results demonstrate the superiority of the proposed approaches. Therefore, the resultant atlases could be applied in related studies. Since our study did not find an optimal cluster number, the cluster number could be set according to one's requirements.

## Author contributions

JW and HW designed the study. JW analyzed the data and drafted the manuscript under the supervision of HW. HW critically revised the manucript. Both authors approved the final version of the manuscript.

### Conflict of interest statement

The authors declare that the research was conducted in the absence of any commercial or financial relationships that could be construed as a potential conflict of interest.
