# Supplementary material for: A Supervoxel-Based Method for Groupwise Whole Brain Parcellation with Resting-State fMRI Data
Source: Front Hum Neurosci. 2016 Dec 27;10:659. doi: 10.3389/fnhum.2016.00659 (PMC5187473; doi:10.3389/fnhum.2016.00659)
Supplement: Supplementary file 1 [file DataSheet1.PDF]

Supplementary Material

A supervoxel-based method for groupwise whole brain parcellation with resting-state fMRI data

Jing Wang[1], Haixian Wang[1*]

[1] Key Laboratory of Child Development and Learning Science of Ministry of Education, Research Center for Learning Science, Southeast University, Nanjing, Jiangsu, PR China

*** Correspondence:**
Dr. Haixian Wang
hxwang@seu.edu.cn

1 Another two sparsifying schemes

Although the weight matrices generated by SS1 and SS2 are closely related to those in (Craddock et al., 2012; Shen et al., 2013), it is meaningful to check if the differences would influence the clustering performances.

The weight matrix in (Craddock et al., 2012) differs from the weight matrix with SS1 by applying a hard threshold 0.5 additionally. Here we denote the sparsifying scheme that combines the spatial constraint and the hard threshold as SS4. With this sparsifying scheme, the five parcellation approaches were applied likewise. The results of different evaluation metrics are shown in Supplementary Figure 3 and Table 3. These results were very close to the results on the weight matrix with SS1. The reason is that the correlations within spatial constraint are generally large, as shown in Figure 1C, thus resisting an additional threshold. Figure 1C also indicates that setting the threshold in a wide range, e.g., a threshold smaller than 0.6, may not greatly affect the clustering performances.

The weight matrix in (Shen et al., 2013) differs from the weight matrix with SS2 by using probably a different sparse rate and a different weighting function. For the first point, we had tried to reserve the 26 rather than 17 largest values in each row and each column of the weight matrix constructed by the Pearson correlation coefficient. This new sparsifying scheme is denoted as SS5. With this sparsifying scheme, the five parcellation approaches were applied likewise. The results of different evaluation metrics are shown in Supplementary Figure 4 and Table 3. The results with SS5 were very similar to those with SS2. Hence, the clustering performances are rather stable with different number of reserved weights. The second point, i.e., using a different weighting function, was examined in the main text of the paper.

2 Computation

Our scripts were written in Matlab(R). The experiments were run on a Dell(R) workstation with twenty 2.80 GHz Intel(R) Xeon(R) processors and 256 GB memory. To reduce computational time, the resting-state fMRI data was downsampled into $4 \times 4 \times 4$ mm^3 resolution, and then a gray matter mask with only 18384 voxels was utilized. By this way, it became manageable to make a full

comparison between different parcellation approaches. During the parcellation procedure, eigendecomposition was the most time-consuming step. In the individual subject level, we calculated the 1000 eigenvectors corresponding to the smallest nontrivial eigenvalues for each subject and each sparsifying scheme only once. In the group level, since we did not employ overclustering by default, much more eigensystems should be solved. The implementation of parallel computing greatly helped to shorten the overall computational time.

Supplementary References

Craddock RC, James GA, Holtzheimer PE, Hu XPP, Mayberg HS. (2012): A whole brain fMRI atlas generated via spatially constrained spectral clustering. Human Brain Mapping 33(8):1914-1928.

Shen X, Tokoglu F, Papademetris X, Constable RT. (2013): Groupwise whole-brain parcellation from resting-state fMRI data for network node identification. Neuroimage 82:403-415.

Xia MR, Wang JH, He Y. (2013): BrainNet Viewer: A Network Visualization Tool for Human Brain Connectomics. Plos One 8(7).

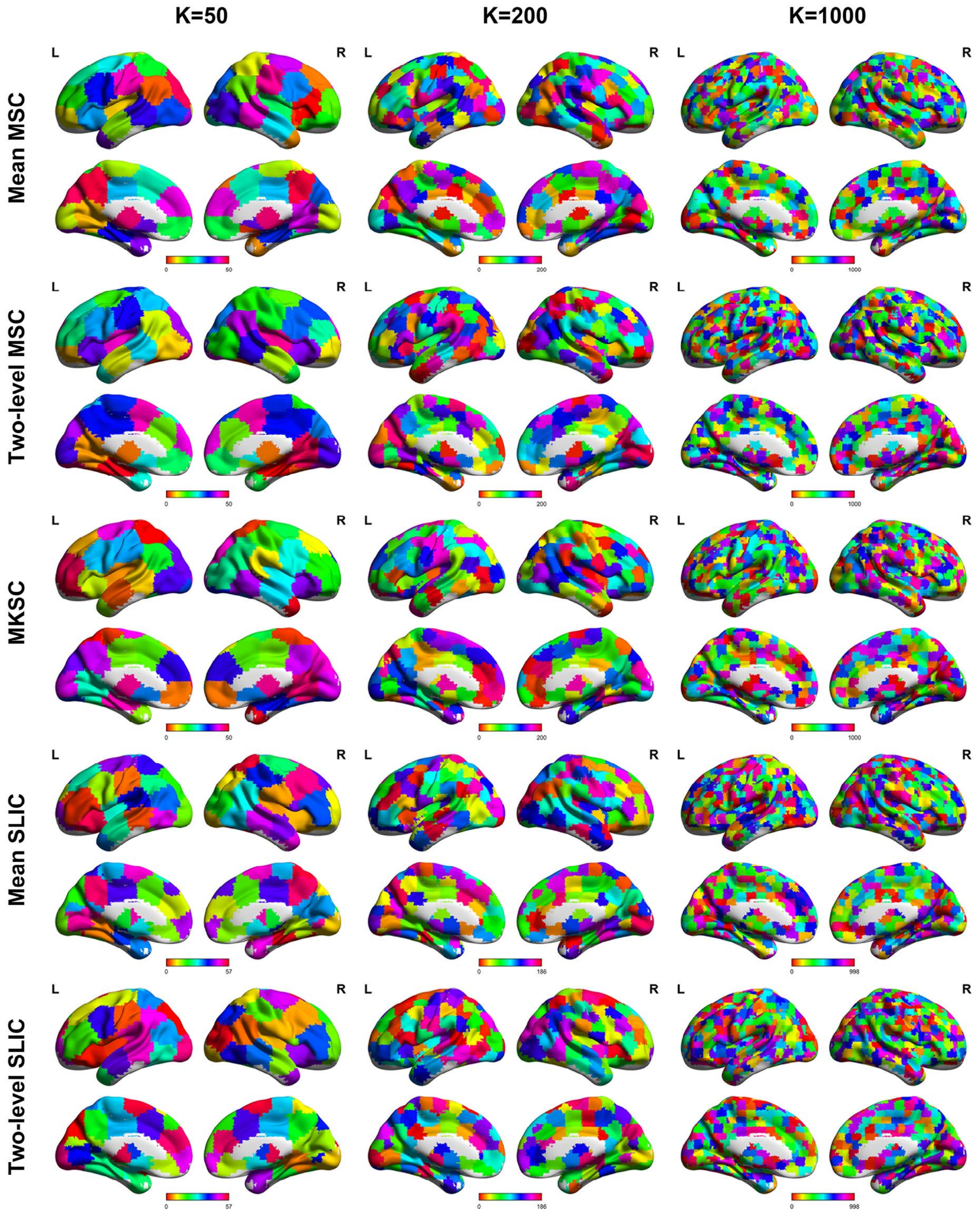

Supplementary Figure 1. Surface mapping of the atlases computed by the five parcellation approaches with SS1 when the cluster number is set to be 50, 200, and 1000. The colormap for each atlas is randomly generated, and each color represents a cluster. The graphs are visualized with the BrainNet Viewer (http://www.nitrc.org/projects/bnv) (Xia et al., 2013).

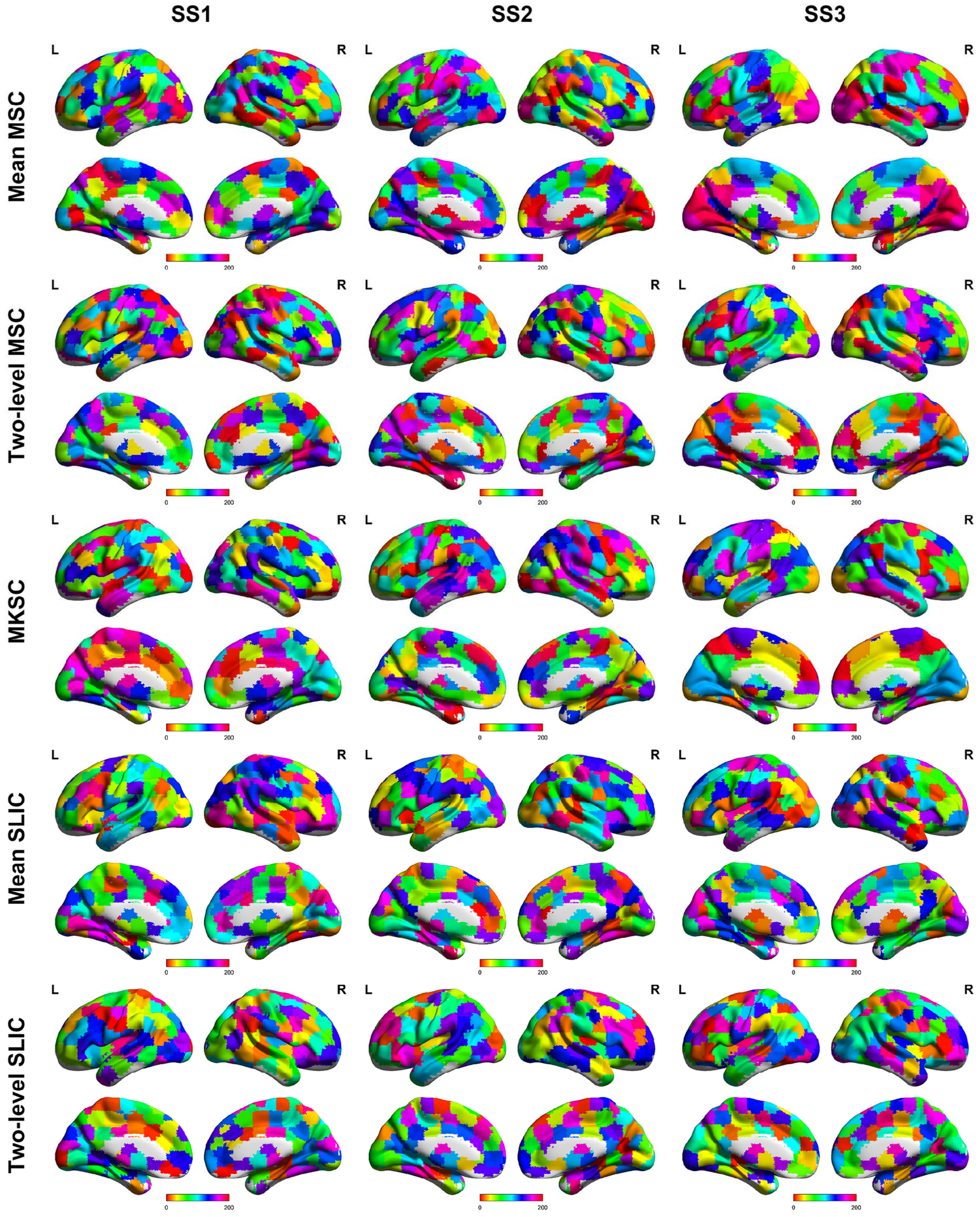

Supplementary Figure 2. Surface mapping of the atlases computed by the five parcellation approaches and the three sparsifying schemes when the initialized cluster number is 200. The colormap for each atlas is randomly generated, and each color represents a cluster. The graphs are visualized with the BrainNet Viewer (http://www.nitrc.org/projects/bnv) (Xia et al., 2013).

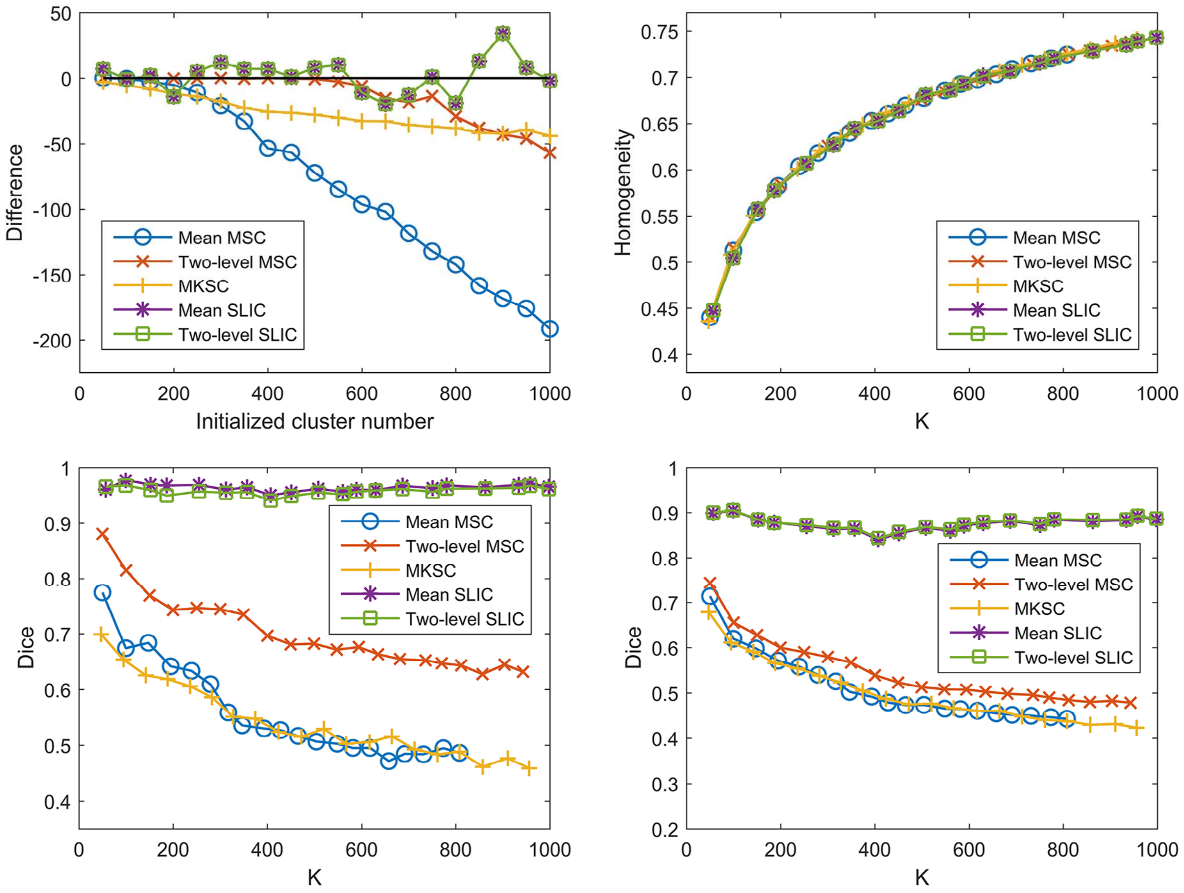

Supplementary Figure 3. The results of different evaluation metrics of the five parcellation approaches when SS4 is employed. The second row shows the results of group-to-group reproducibility and group-to-subject reproducibility from left to right.

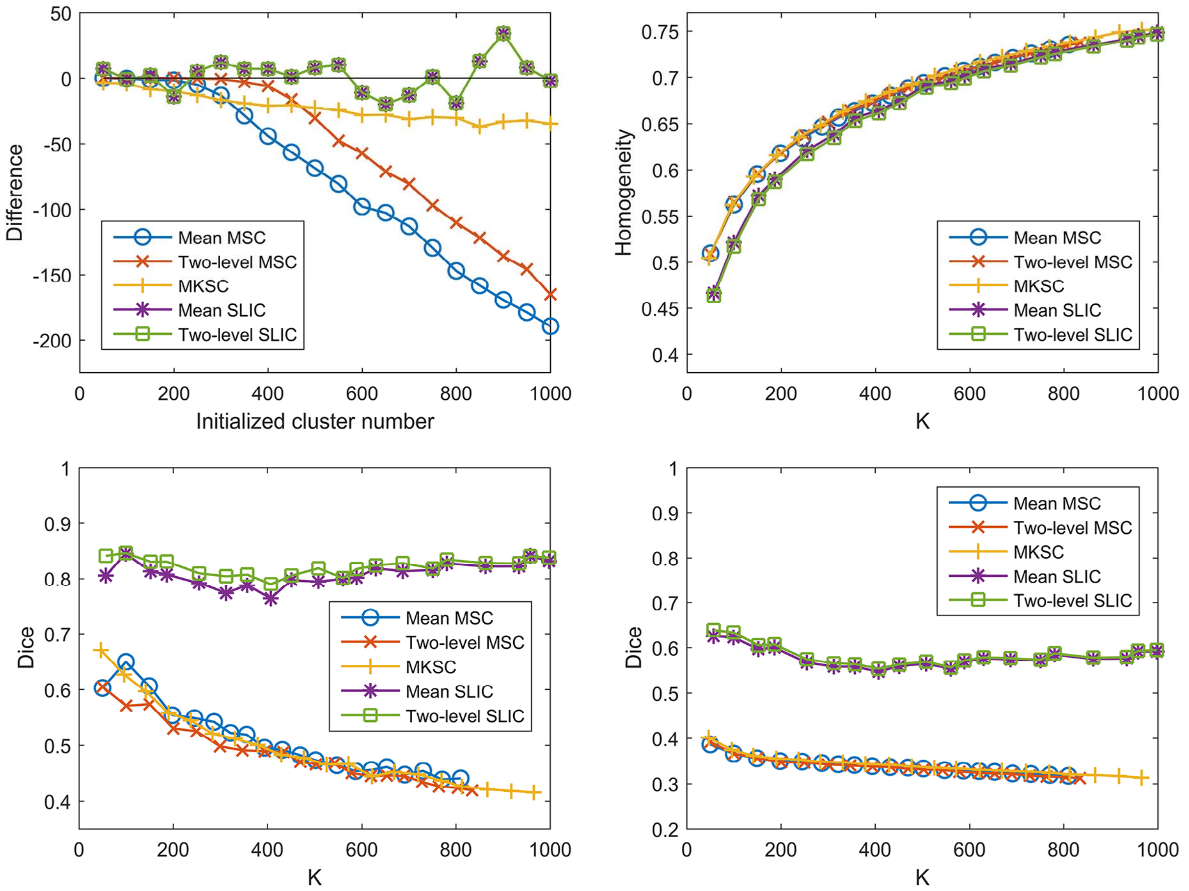

Supplementary Figure 4. The results of different evaluation metrics of the five parcellation approaches when SS5 is employed. The second row shows the results of group-to-group reproducibility and group-to-subject reproducibility from left to right.
